# Differential substrate degradation by super-degron: EGFP in wild-type mouse cells, PD-1 requires CRBN humanization

**DOI:** 10.1016/j.isci.2025.112992

**Published:** 2025-06-23

**Authors:** Chie Naruse, Ojiro Ishibashi, Masatoshi Ohgushi, Hirohiko Imai, Tomoko Matsuzaki, Xuchi Pan, Tatsuhiko Miyazaki, Yuka Shidahara, Yu Shirakawa, Fumihiro Sugiyama, Masahide Asano

**Affiliations:** 1 Institute of Laboratory Animals, Graduate School of Medicine, Kyoto University, Kyoto 606-8501, Japan; 2 Laboratory of Developmental Systems, Department of Biosystems Science, Institute for Life and Medical Sciences, Kyoto University, Kyoto 606-8507, Japan; 3 Department of Informatics, Graduate School of informatics, Kyoto University, Kyoto 606-8501, Japan; 4 Innovation Research Center for Quantum Medicine, Gifu University Hospital, Gifu 501-1194, Japan; 5 Department of Pathology, Gifu University Hospital, Gifu 501-1194, Japan; 6 Bioscience Business Division, KAC Co., Ltd. Ritto, Shiga 520-3001, Japan; 7 Laborarory Animal Resource Center, Transborder Medical Research Center, Institute of Medicine, University of Tsukuba, Tsukuba 305-8575, Japan

**Keywords:** Natural sciences, Biological sciences, Immunology

## Abstract

Protein knockdown using a zinc finger degron tag and thalidomide analogs was previously considered ineffective in mouse cells. However, using EGFP as an indicator, we found that a super-degron tag (SD) enables degradation in mouse cells when combined with iberdomide or mezigdomide. While SD-tagged EGFP was degraded in wild-type mouse cells, SD-tagged PD-1 required human-type CEREBLON (CRBN^I391V^) for degradation. In mice with CRBN^I391V^, endogenous PD-1 tagged with SD was efficiently degraded in T cells both *in vitro* and *in vivo*. Compared with anti-PD-1 antibody treatment, the degradation of PD-1 led to more rapid activation of CD8^+^ T cells. Moreover, pomalidomide that crosses the brain-blood barrier reduced PD-1 expression in the brain. These results suggest that SD and thalidomide analogs can be used for *in vitro* and *in vivo* protein knockdown in mice, although some conditional settings are required.

## Highlights

• Iberdomide induced degradation of SD-tagged EGFP in mouse, rat, and human cells
• CRBN humanization enhanced degradation efficiency by 10-100 fold in mouse cells
• Unlike EGFP, SD-tagged PD-1 was not degraded in wild-type mouse cells
• *In vivo*, iberdomide suppressed MC-38 tumor growth faster than an antibody by degrading SD-tagged PD-1

## Graphical abstract

**Figure:**
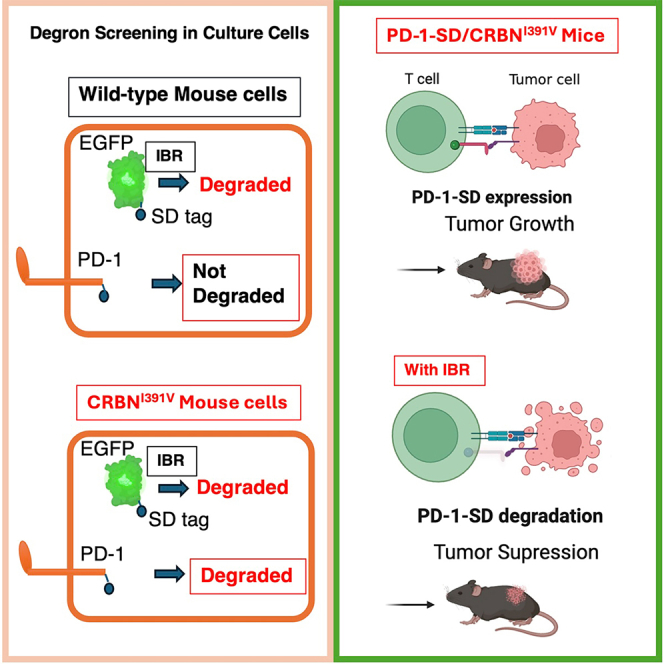

## Introduction

The proteasome system is essential for cellular maintenance via protein degradation. The proteasome not only degrades unwanted proteins, such as misfolded or overexpressed proteins, but also precisely regulates the cell cycle, signal transduction, and cellular responses, ensuring the maintenance of the organism’s health.^1^^,^^2^^,^^3^ Proteins targeted for degradation are polyubiquitinated by E3 ubiquitin ligase, recognized by ubiquitin-dependent chaperones, and transported to the proteasome.^4^^,^^5^^,^^6^ Recently, there have been made to hijack the proteasome-dependent proteolysis inherent in cells to degrade disease-causing proteins. Based on the idea that one end of a degradation target binds to a small molecule or peptide and the other end binds to an E3 ubiquitin ligase, the degradation target is ubiquitinated and degraded, and proteolysis-targeting chimera (PROTAC) is being actively developed as a drug to treat diseases by degrading disease-causing molecules.^7^^,^^8^^,^^9^

Unlike gene knockout or mRNA degradation, the analysis of protein function by the protein degron system is reversible, and the effect appears more quickly than with RNA interference method. In addition, because the amount of reduction in protein expression can be controlled, it is expected to be applied not only to disease treatment but also to basic research. Although it is not easy for individual researchers to synthesize small molecules or peptides, such as PROTAC, for every protein they study, it is relatively easy to incorporate a proteolytic tag, called a degron tag, into target genes by genome engineering. Several degron tags have been developed for use in cells and *in vivo*, enabling the targeted degradation of endogenous proteins through the addition of drugs or small molecules.^10^^,^^11^ The auxin-degron, dTAG, and Small-Molecule-Assisted Shutoff (SMASh) tags have been added to endogenous proteins by genome editing and can be used for whole-body protein degradation in mice.^12^^,^^13^^,^^14^ However, several challenges persist for *in vivo* application, including the large size of the tag, which makes genome editing in mice difficult, and the high cost of small molecules required for degradation in mice.

One of the smallest tags is the Ikaros family zinc finger protein (IKZF) tag, which binds to the CEREBLON (CRBN) E3 ubiquitin ligase via a thalidomide analog. The target protein tagged with the zinc finger domain in IKZF1/3 is degraded by the addition of a thalidomide analog in human cells.^15^ CRBN is involved in the ubiquitination of proteins such as Myeloid Ecotropic Viral Integration Site 2 (MEIS2), which plays an important role in development and leukemogenesis.^16^^,^^17^ MEIS2 is one of the endogenous targets that biochemical screening has shown to be ubiquitinated by CRBN.^18^ However, when thalidomide analogs are added, human CRBN binds strongly to proteins such as IKZF1, IKZF3, and Casein Kinase 1 alpha 1 (CK1a), resulting in their degradation. The transcription factor P63, essential for human fetal limb development and heart formation, has been identified as a new substrate for CRBN. SALL4 is also responsible for thalidomide-induced teratogenicity in humans and is degraded when thalidomide interacts with CRBN.^19^ Consequently, thalidomide administration to pregnant women results in fetal limb dysplasia and heart malformations.^20^ However, in mice and rats, these new substrates are not degraded in the presence of thalidomide derivatives due to differences in the amino acid sequence of CRBN between humans and rodents.^21^^,^^22^^,^^23^ Thalidomide administration to mice with human-type CRBN, in which the 391^st^ isoleucine (Ile) of mouse CRBN is replaced by a valine (Val) (CRBN^I391V^), results in the degradation of the new substrate and causes partial abnormalities in limb development.^24^^,^^25^ Recently, the super-degron tag (SD), featuring a chimeric structure of the IKZF1/3 common zinc finger domain and the ZFP91 zinc finger domain, has emerged as a promising candidate for protein degradation in human cells transplanted into mice^26^^,^^27^; however, knockdown of endogenous proteins in mice using this approach has not yet been attempted.

Previously we attempted to knock down endogenous PD-1 in mice and found that the use of the SMASh tag resulted in significant leakage, inefficient knockdown, and prolonged knockdown duration.^14^ In this study, we found that EGFP added with an SD in its C-terminus was degraded by the addition of iberdomide (IBR) not only in *Crbn*^I391V/I391V^ mouse cells but also in wild-type mouse cells. Despite the efficient degradation of EGFP in wild-type mouse cells, PD-1 degradation in wild-type mouse cells showed significant leakage and inefficiency. For *in vivo* knockdown, we examined the effect of the SD on endogenous PD-1 expression in *Crbn*^I391V/I391V^ mice. The proliferation of MC-38 adenocarcinoma cells was suppressed by the system *in vivo*, suggesting that the knockdown system can be used *in vivo* in mice. The degron system also made it possible to compare PD-1 repression by antibodies with the reversible knockdown by SD. We also evaluated *in vivo* effects of PD-1 degradation in the brain, where protein degradation using small molecules is difficult due to the blood-brain barrier. These results indicate that SD is useful for knocking down endogenous proteins in mouse cells and mice. However, the conditions must be considered for each protein of interest.

## Results

### Degradation of PD-1 in the human Jurkat cell line was successful with the super-degron tag but not with the other tags

**Figure 1:**
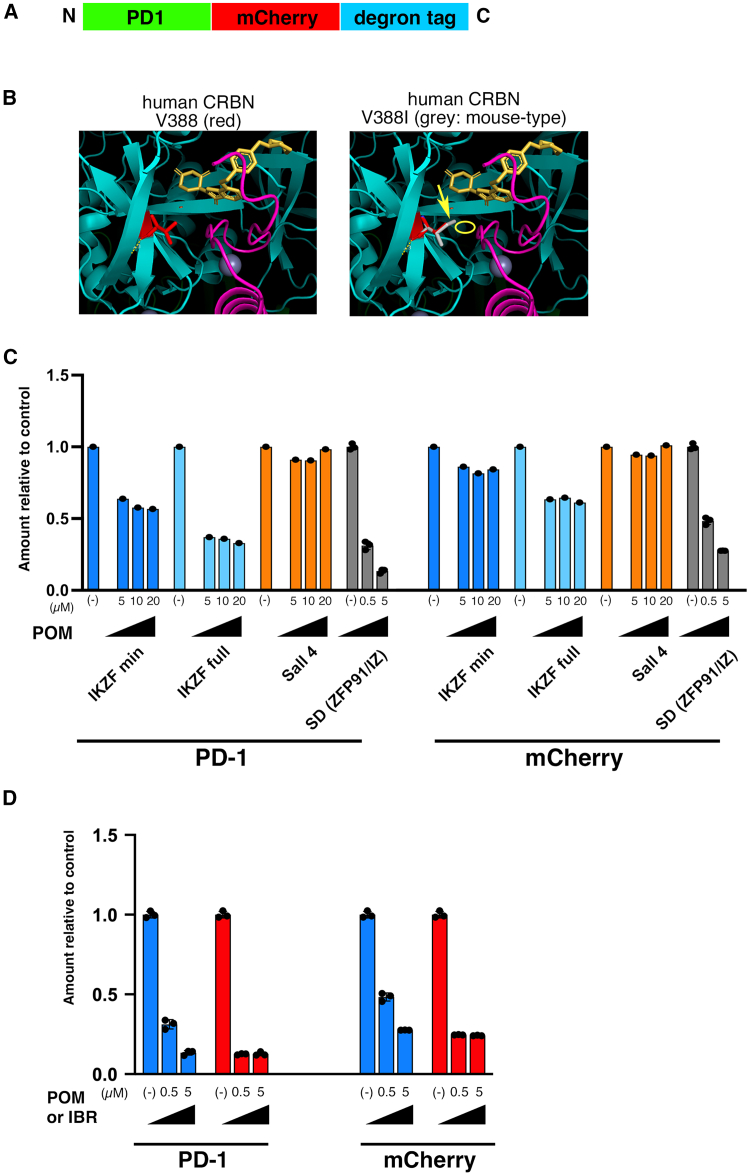
Degradation of various degron-tagged mouse PD-1 in human Jurkat cell line (A) The protein construction of PD-1 tagged with mCherry and a degron tag. (B) The partial structure of human and mouse CRBN critical for binding among CRBN (blue), zinc finger domain of IKZF1/3 (pink) and Iberdomide (IBR, yellow). Valine 388 in human CRBN (red on the left) is replaced by isoleucine in mouse CRBN (gray on the right, over the red valine), causing a conflict between CRBN and IKZF1/3 (yellow ellipse). The stretch of the methyl group in isoleucine causing the conflict is indicated by a yellow arrow. (C) The amount of PD-1 and mCherry fused to IKZF minimal (blue, *n* = 1), IKZF full length (light blue, *n* = 1), Sall4 (orange, *n* = 1) or super degron (SD; red, *n* = 3) tags in Jurkat human T cell lymphomas 24 h after the addition of pomalidomide (POM) measured by flow cytometry. (D) The amount of PD-1 and mCherry fused to SD tag in Jurkat cells 24 h after the addition of pomalidomide (POM; blue, *n* = 3) or iberdomide (IBR; red, *n* = 3) measured by flow cytometry. (C and D) The amount was standardized with the sample to which no reagent was added as 1. Concentrations of reagents were 0 (−), 5, 10, and 20 μM for IKZF minimal, IKZF full-length, and Sall4 tags and 0 (−), 0.5, 5 μM for SD tag. The dots indicate the results of the experiment for each of samples. Data are represented as mean ± standard deviation when *n* = 3.

To identify a tag that could efficiently knock down mouse PD-1, we connected various degron tags to the C-terminus of PD-1, including IKZF1/3, Sall4, ZFP91, and SD, and generated Jurkat human T cell lymphoma cell lines that stably expressed the mouse PD-1-degron tag (Figure 1A). As reported previously,^21^^,^^22^^,^^23^ in mouse CRBN, the 391^st^ amino acid corresponding to Val-388 of human CRBN is Ile, which causes a protrusion when a *neo* substrate binds to CRBN, resulting in a weaker affinity than in human CRBN (Figure 1B). Therefore, a human cell line was used because it was thought that mouse CRBN would not degrade SD by thalidomide analogs. PD-1 expression on the cell surface can be analyzed by flow cytometry using an anti-mouse PD-1 antibody, which does not recognize human PD-1. We also analyzed the degradation of total proteins using mCherry.

PD-1 expression on the cell surface with the SD was greatly reduced by the addition of pomalidomide (POM) (0.11 times of control, Figure 1C; Table S1). In contrast, the expression levels of the PD-1-IKZF minimal tag and PD-1-IKZF full-length tag on the cell surface after the addition of POM were only 0.53 and 0.32 times that of the control, respectively. The Sall4 tag did not reduce protein levels in this study. We also used IBR to degrade PD-1-SD because CRBN E3 ligase modulatory drugs (CELMoDs, including IBR) degrade neosubstrates more efficiently than immunomodulatory drugs (IMiDs, including POM). 5 μM POM and 0.5 μM IBR showed the same effect (Figure 1D; Table S1). The decrease in mCherry was smaller than that of PD-1 24 h after the drug was added (Figures 1C and 1D; Table S1), suggesting that proteins inside the cell might be degraded more slowly or need more reagent than those on the cell surface.

### Super-degron tag induced the degradation of EGFP by the addition of ImiDs or CELMoDs in wild-type mouse cells *in vitro*

**Figure 2:**
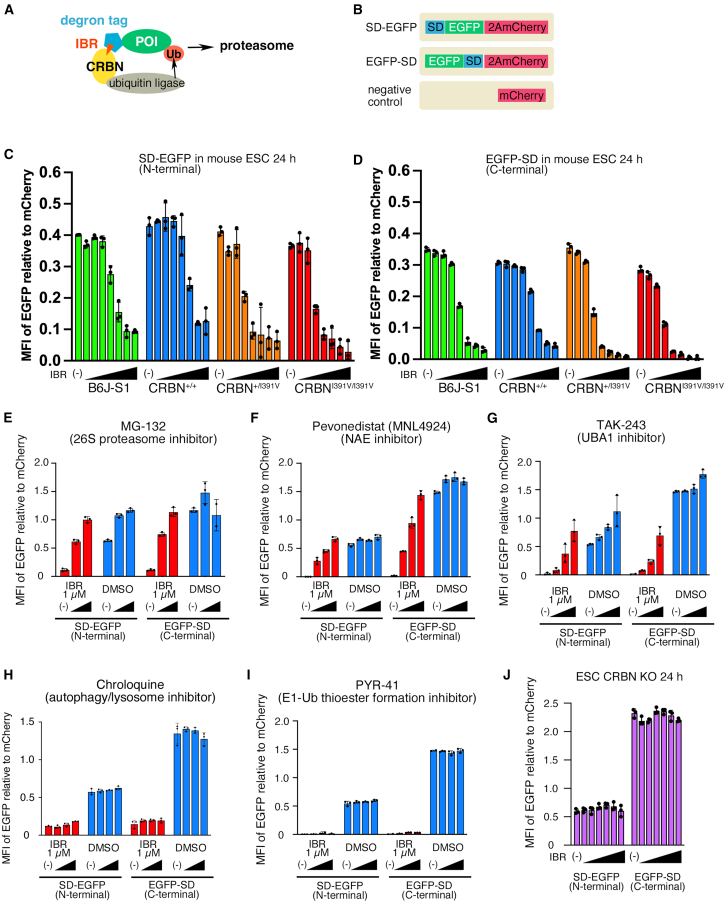
Comparison of the degradation of Super degron-tagged EGFP in mouse embryonic stem cells with the addition of iberdomide (A) The scheme of the CRBN-dependent degradation of the super degron (SD)-tagged protein. IBR: iberdomide, POI: protein of interest, Ub: ubiquitin, SD: super degron tag. (B) The protein-producing vectors used in Figure 2. Top; N-terminal SD vector (SD-EGFP vector; CAG-SD-EGFP-2A-mCherry-2A-Neo-pA), middle; C-terminal SD vector (EGFP-SD vector; CAG-EGFP-SD-2A-mCherry-2A-Neo-pA), bottom; negative control vector (CAG-mCherry). (C and D) The amount of SD-EGFP (C) or EGFP-SD (D) in mouse embryonic stem cells (ESCs) (green: B6J-S1, blue: CRBN^+/+^, orange: CRBN^+/I391V^, red: CRBN^I391V/I391V^) 24 h after the transfection of ESCs with the SD tagged EGFP-expressing vectors and addition of 1 μM iberdomide (IBR). (E-I) Inhibition of the degradation of SD-EGFP or EGFP-SD in B6J-S1 by MG-132 (26S proteasome inhibitor, (E), pevonedistat (NAE inhibitor, (F), TAK-243 (NAE inhibitor, (G), chloroquine (lysosome and autophagy inhibitor, (H) and PYR-41 (E1-Ub thioester formation inhibitor, (I). (J) The amount of SD-EGFP or EGFP-SD in *Crbn*^−/−^ (CRBN KO) mouse ESCs 24 h after transfection. (C–J) Reagents was added immediately after transfection. The triangles indicate the increasing concentration of reagents. The reagent concentration is 1 μM (C, D and J: IBR), 4 μM (E–G: MG-132, pevonedistat, and TAK-243, respectively), 1 μM (H: chroloquine), and 100 μM (I: PYR-41) in the rightmost bar in each group, and the left neighbor is a step dilution of one-tenth (C, D, H, and J) and one-half (E-G and I). The concentration of IBR in E-I is fixed at 1 μM. Intensities of EGFP and mCherry in mCherry positive cells were measured by flow cytometry, and the geometric means of fluorescence intensities (MFI) were calculated. The average of the MFI of the EGFP in the negative control was subtracted from each MFI of EGFP, and the value of each sample was divided by the mCherry MFI of each sample. The dots indicate the results of the experiment for each of the three samples. Data are represented as mean ± standard deviation.

To apply the CRBN-IMiD-based degron system to mouse whole body (Figure 2A), CRBN-humanized mice were generated using genome editing (Figure S1). To compare the degradation of super-degron-tagged EGFP in mouse cells, we generated wild-type (*Crbn*^+/+^), heterozygous (*Crbn*^+/I391V^), and homozygous (*Crbn*^I391V/I391V^) mouse embryonic stem cells (mESCs). We compared the degradation of transiently expressed super-degron (SD)-EGFP (N-terminal SD) and EGFP-SD (C-terminal SD) (Figure 2B) in *Crbn*^+/+^, *Crbn*^+/I391V^ and *Crbn*^I391V/I391V^ mESCs. Both SD-EGFP and EGFP-SD were degraded by IBR at almost the same concentration of half-maximal degradation (DC_50_) (Table S2). For example, in *Crbn*^I391V/I391V^ mESCs, DC_50_s of both SD-EGFP and EGFP-SD were 10^−4.2^ μM. The predicted amount of EGFP at the highest degradation with increasing IBR concentrations was 0.13– to 0.24-fold of the initial expression level with SD-EGFP, to 0.03– to 0.15-fold with EGFP-SD, suggesting that the C-terminal SD was more effective.

Unexpectedly, SD-EGFP and EGFP-SD were reduced with the addition of IBR, not only in *Crbn*^+/I391V^ and *Crbn*^I391V/I391V^ mESCs but also in *Crbn*^+/+^ mESCs (Figures 2C, 2D, and S2). When the IBR concentration was increased, the amount of EGFP was reduced to 0.25- (SD-EGFP) and 0.15- (EGFP-SD) fold of the initial amount in *Crbn*^+/+^ mESCs without IBR, although the DC_50_ of *Crbn*^+/+^ mESCs was approximately 10-fold higher than that of *Crbn*^+/I391V^ and *Crbn*^I391V/I391V^ mESCs (Table S2). Because *Crbn*^+/+^ mESCs were newly generated in our laboratory, their characteristics may differ from those of the conventional mESC line. Therefore, we performed the same experiment using B6J-S1 cells, an mESC line derived from C57BL/6,^28^ and observed SD-EGFP and EGFP-SD degradation in B6J-S1 cells, similar to that observed in *Crbn*^+/+^ mESCs (Figures 2C and 2D; Table S2). DC_50_ showed a little difference between B6J-S1 and *Crbn*^+/+^ ESCs (SD-EGFP: 10^−2.7^ μM and 10^−2.2^ μM, respectively; EGFP-SD: 10^−9.1^ μM and 10^−8.7^ μM, respectively). The DC_50_ values of *Crbn*^+/I391V^ and *Crbn*^I391V/I391V^ mESCs were similar.

We examined whether the degradation observed in wild-type mouse cells was proteasome- or lysosome-dependent by adding inhibitors to each cell line. EGFP degradation was inhibited in a concentration-dependent manner by MG-132 (proteasome inhibitor, Figure 1E), MNL4924 (NAE inhibitor, Figure 2F), and TAK-243 (UBA1 inhibitor, Figure 2G), but not by chloroquine (autophagy/lysosome inhibitor, Figure 2H), suggesting that the proteolysis observed in mouse cells was proteasome-dependent. PYR-41 (E1-Ub thioester formation inhibitor, Figure 2I) did not affect the degradation of EGFP, suggesting that the pool of E1-Ub was sufficient for the degradation of EGFP in this study or that there might be some supplemental systems besides E1-Ub. To determine whether this degradation occurred in a CRBN-dependent manner, we introduced vectors into *Crbn*^−/−^ (CRBN-KO) mESCs (Figure S3) and examined EGFP degradation. Degradation of SD-tagged EGFP did not occur in CRBN-KO mESCs (Figure 2J), suggesting that the degradation of SD-tagged EGFP by IBR is CRBN-dependent.

### SD-tagged EGFP could be degraded by thalidomide analogs in various wild-type mouse cell lines

**Figure 3:**
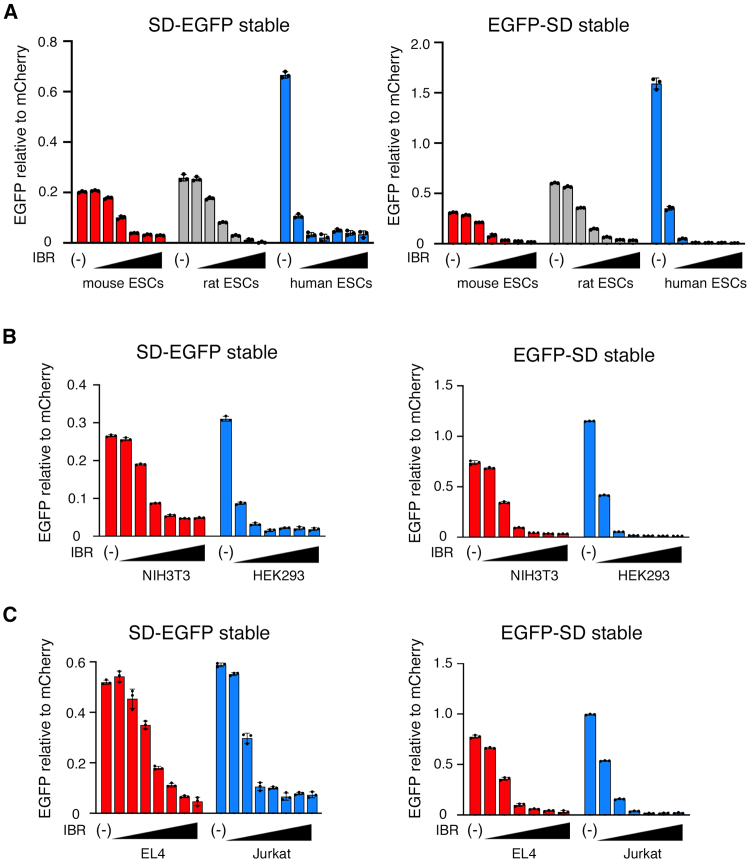
Comparison of the degradation of super degron tagged-EGFP in human and mouse cell lines with the addition of iberdomide The amount of SD-EGFP or EGFP-SD in mouse, rat, and human embryonic stem cells (ESCs) (A), mouse NIH3T3 embryonic fibroblast cell line and human HEK293 cell line (B), and mouse EL4 T cell lymphoma cell line and human Jurkat T cell leukemia cell line (C) stably expressing transgenes. The triangles indicate the increasing concentration of IBR. (A–C) IBR concentrations are 1 μM in the rightmost bar of each group and one-tenth step dilution in the left neighbor; EGFP intensity measurements and analyses are the same as in Figure 2. The dots indicate the experimental results for each of the three samples. Data are represented as mean ± standard deviation.

Next, we compared the degradation of stably expressed SD-tagged EGFP in ESCs of various species. SD-EGFP and EGFP-SD were also degraded in rat ESCs (rESCs) with CRBN, in which the amino acid corresponding to the 391^st^ isoleucine in mice is isoleucine, as in mice (Figure 3A; Table S3). The DC_50_ of human ESCs (hESCs) was approximately 100-fold higher (10^−5.9^ μM) than mESCs (10^−3.2^ μM) and rESCs (10^−3.6^ μM) (Figure 3A; Table S3). Furthermore, the relative expression of SD-EGFP and EGFP-SD to standard mCherry expression in mESCs (0.20 and 0.30, respectively) and rESCs (0.26 and 0.60, respectively) without IBR was lower than hESCs (0.67 and 1.59, respectively), suggesting that degradation leakage in drug-free mESCs and rESCs may be greater than that in hESCs. However, in mESCs and rESCs, higher concentrations of IBR (1 μM) ultimately reduced protein content to human levels. SD-EGFP was reduced by 0.146-fold in mESCs and 0.057-fold in rESCs, whereas 0.051-fold in hESCs compared to controls. EGFP-SD was reduced by 0.085-fold mESCs and 0.083-fold in rESCs, whereas 0.005-fold in hESCs compared to controls.

The degradation activity of ESCs is higher than that of other cells.^29^ To examine whether degradation in mESCs was due to cell-specific metabolism in ESCs, we performed similar experiments in other mouse cell lines. Comparing the degradation of SD-tagged EGFP in mouse NIH/3T3 embryonic fibroblasts and EL4 T cell lymphoma cell lines with that in human HEK293 and Jurkat cell lines, degradation occurred in both mouse NIH/3T3 and EL4 cells (Figures 3B and 3C). In NIH/3T3 cells, DC_50_ of SD-EGFP and EGFP-SD were 10^−3.7^ μM and 10^−4.1^ μM, whereas 10^−5.5^ μM and 10^−5.3^ μM in HEK293 cells, respectively. In EL4 cells, DC_50_ of SD-EGFP and EGFP-SD were 10^−3.7^ μM and 10^−4.1^ μM, whereas 10^−5.1^ μM and 10^−4.9^ μM in Jurkat cells, respectively (Figures 3B and 3C; Table S3). Similar to ESCs, leakage in mouse cell lines may be greater than that in human cell lines, although the difference was less than that in ESCs. Without IBR, the expression of SD-EGFP and EGFP-SD relative to the standard mCherry expression in NIH/3T3 cells (0.27 and 0.75, respectively) and EL4 cells (0.51 and 0.76, respectively) was lower than that in HEK293 cells (0.31 and 1.2, respectively) and Jurkat cells (0.60 and 0.99, respectively). However, in mouse cell lines, higher concentrations of IBR (1 μM) reduced EGFP to human-levels such as ESCs. SD-EGFP was reduced by 0.19-fold in NIH/3T3 cells and 0.15-fold in EL4 cells, whereas it was reduced by 0.065-fold in HEK293 cells and 0.128-fold in Jurkat cells compared to controls. EGFP-SD expression was reduced by 0.049-fold in NIH/3T3 cells and 0.057-fold in EL4 cells, whereas it was reduced by 0.009-fold in HEK293 cells and 0.024-fold in control cells. These results indicate that SD-tagged proteins are efficiently degraded by the addition of IBR in a variety of mouse cells, although less efficiently than in human cells.

### Rapid degradation and recovery of SD-tagged EGFP using appropriate dose of iberdomide

**Figure 4:**
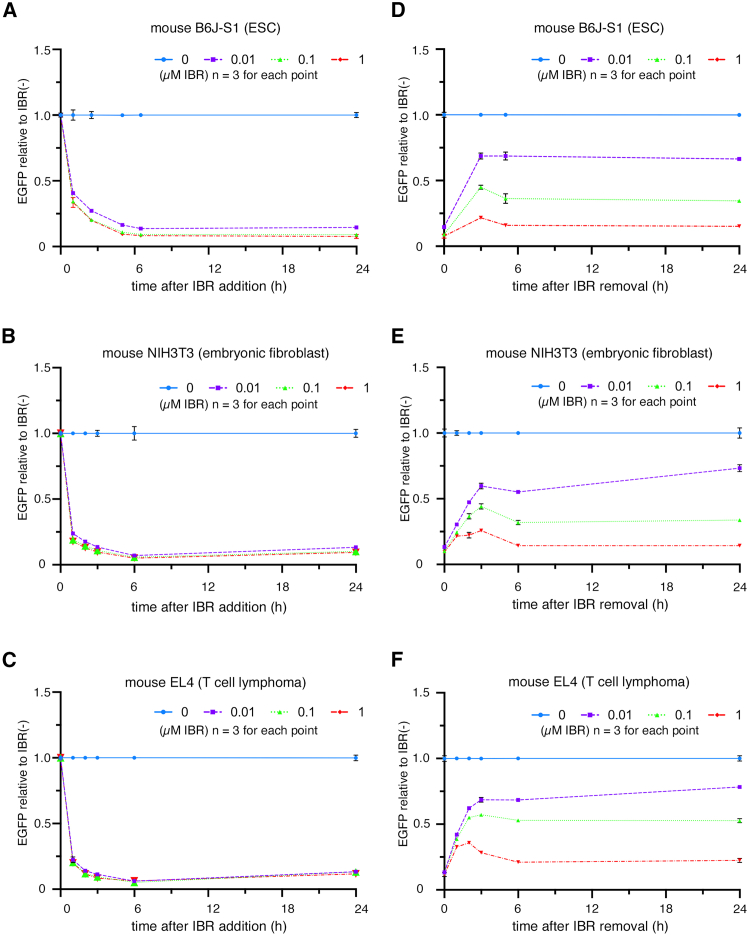
Time course of degradation and recovery of Super degron-tagged EGFP in mouse cell lines by the addition of iberdomide (A–F) The degradation (A–C) and recovery (D–F) time course of EGFP-SD in B6J-S1 mouse embryonic stem cell line (A and D), NIH3T3 mouse embryonic fibroblast cell line (B and E), and EL4 mouse T cell lymphoma cell line (C and F) stably expressing transgenes. EGFP intensity measurements and analyses are the same as in Figure 2. The dot and bar show the mean and error bars show standard deviations of the experiment for each time point of the three samples, although some error bars are too short to be visible.

The degradation and recovery time courses of EGFP-SD were analyzed using stably transfected mouse cell lines (Figure 4; Table S4). The half-lives (T_1/2_) of EGFP-SD after the addition of 0.01 μM IBR in mESCs, NIH/3T3 cells, and EL4 cells were 37.5, 21.7, and 21.0 min, respectively (Figures 4A–4C; Table S4). In each cell line, IBR concentrations of 0.01–1 μM showed little difference in T_1/2_, although degradation was slightly faster at higher concentrations. The EGFP-SD expression was minimal after 6 h. In mESCs, NIH/3T3 cells and EL4 cells, the minimal levels of EGFP-SD after the addition of 0.01 μM IBR were 0.16-, 0.12- and 0.11-fold of the control without IBR, respectively.

The recovery time of EGFP-SD after IBR removal was also examined in mESCs, NIH/3T3 cells, and EL4 cells (Figures 4D–4F). EGFP-SD expression levels rapidly increased to approximately 70% of initial levels at 3 h after removal of 0.01 μM IBR and were finally recovered more than 60% of the initial level at 24 h after 0.01 μM IBR removal (0.66-, 0.73-, and 0.78-fold in mESCs, NIH/3T3 cells, and EL4 cells, respectively, compared to controls). Unexpectedly, EGFP-SD expression levels increased once at 3 h after removal of IBR and then decreased again at 6 h when 0.1 and 1 μM of IBR were added and removed. Considering the possibility that IBR removal was insufficient or that the IBR remaining in the cells might have been released into the medium and affected other cells, the cells were washed and cultured again 2 h after the first removal. However, the expression levels of EGFP-SD at 6 and 24 h were similar to those observed after a single wash (data not shown). Considering the balance between reduction in protein content and recovery, 0.1 to 0.01 μM IBR may be optimal for cultured mouse cells.

### Degradation of SD-tagged EGFP occurred with other thalidomide analogs

**Figure 5:**
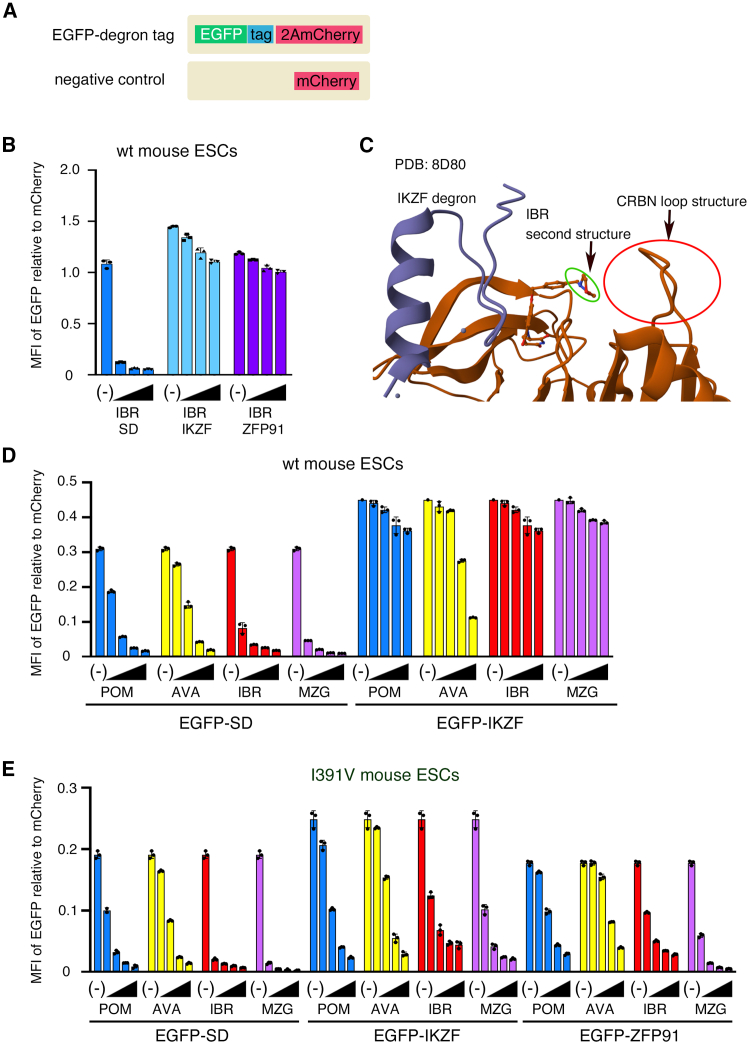
Degradation of degron-tagged EGFP in mouse embryonic stem cells by the addition of pomalidomide, avadomide, iberdomide, and mezigdomide (A) The protein-producing vectors used in Figure 5. Upper; C-terminal-degron tag vectors (EGFP- degron tag vector; CAG-EGFP-degron tag-2A-mCherry-2A-Neo-pA), lower; negative control vector (CAG-mCherry). tag: degron tag (SD, IKZF or ZFP91). (B) The amount of EGFP-SD, EGFP-IKZF-, and EGFP-ZFP91 with the addition of iberdomide (IBR) in B6J-S1 mouse embryonic stem cells (mESCs). (C) The structure of IKZF degron tag, IBR, and human CRBN. The data is from the Protein DataBank (https://www.rcsb.org/?ref=nav_home) and the ID number is PDB: 8D80. Red circle: the essential structure of CRBN for binding to IBR. Green circle: a part of the second structure of IBR for binding the loop of CRBN. (D) The amount of EGFP-SD and EGFP-IKZF in B6J-S1 mESCs with pomalidomide (POM), avadomide (AVA), IBR, and mezigdomide (MZG). (E) The amount of EGFP-SD, EGFP-IKZF, and EGFP-ZFP91 in *Crbn*^I391V/I391V^ mESCs with POM, AVA, IBR, and MZG. (B, D, and E) Reagents or solvent (−) were added to the mESCs stably expressing transgenes. The triangles indicate the concentration of reagents. The concentration is 1 μM in the rightmost bar in each group, and the left neighbor is a step dilution of one-tenth. EGFP intensity measurements and analyses are the same as in Figure 2. The dots indicate the results of the experiment for each of the three samples. Data are represented as mean ± standard deviation.

Although SD is a chimeric molecule of the zinc fingers of IKZF1/3 and ZFP91, its degradation efficiency in human cells is higher than that of the IKZF1/3 and ZFP91 degron tags. However, combinations of these tags and thalidomide analogs have not been precisely tested for protein degradation in mouse cells. Therefore, the degradation efficiencies of EGFP-SD, EGFP-IKZF, and EGFP-ZFP91 stably expressed in mESCs were reexamined using flow cytometry (Figure 5A). EGFP-IKZF and EGFP-ZFP91 were degraded much less by IBR than SD (Figure 5B).

Next, the relationship between the tags and drugs was investigated (Figure 5D; Table S5). When comparing the changes in EGFP-IKZF and EGFP-ZFP91 expression with and without IBR, EGFP-IKZF showed a slightly higher percentage decrease (Figure 5B). Therefore, SD and IKZF tags, but not the ZFP91 tag, were further investigated. Pomalidomide (POM) was used as a representative IMiD, while avadomide (AVA), IBR, and mezigdomide (MZG) were used as representatives of CELMoDs. Among these, IBR and MZG have secondary structures that stabilize their binding to the loop structure of CRBN, and their affinity is stronger than that of IMiDs^30^ (Figure 5C). As expected, EGFP-SD was more efficiently degraded by IBR (DC_50_:10^−3.6^ μM) and MZG (DC_50_:10^−3.9^ μM) than by POM (DC_50_:10^−2.9^ μM) and AVA (DC_50_:10^−2.1^ μM) (Figure 5D; Table S5). In contrast, EGFP-IKZF was not effectively degraded by POM, IBR, or MZG (DC_50_:10^−1.6^, 10^−1.6^, and 10^−2.0^ μM, respectively). Unexpectedly, EGFP-IKZF was significantly reduced in mESCs with 0.1 and 1 μM of AVA compared to other reagents, probably due to its structural affinity for mouse CRBN. However, the efficiency was very low (DC_50_:10^−0.90^ μM) compared to EGFP-SD (DC_50_:10^−2.1^ μM).

We also compared the effects of these reagents on the expression of EGFP-SD, EGFP-IKZF, and EGFP-ZFP91 in *Crbn*^I391V/I391V^ mESCs (Figure 5E; Table S5). IBR (DC_50_:10^−4.2^ μM) and MZG (DC_50_:10^−4.2^ μM) degraded EGFP-SD more efficiently than POM (DC_50_:10^−3.0^ μM) and AVA (DC_50_:10^−2.2^) in *Crbn*^I391V/I391V^ mESCs. The efficiency of EGFP-IKZF degradation in *Crbn*^I391V/I391V^ mESCs was significantly higher than that in *Crbn*^+/+^ mESCs. EGFP-ZFP91 was efficiently degraded by IBR and MZG in *Crbn*^I391V/I391V^ mESCs. However, the IKZF and ZFP91 tags degraded at a lower efficiency than the SD with any reagent. Therefore, we determined that a combination of the SD and IBR or MZG may be suitable for protein degradation in mouse cells.

### Endogenous PD-1 tagged with SD showed high efficiency of degradation in *Crbn*^I391V/I391V^, but not in *Crbn*^+/+^ T cells

**Figure 6:**
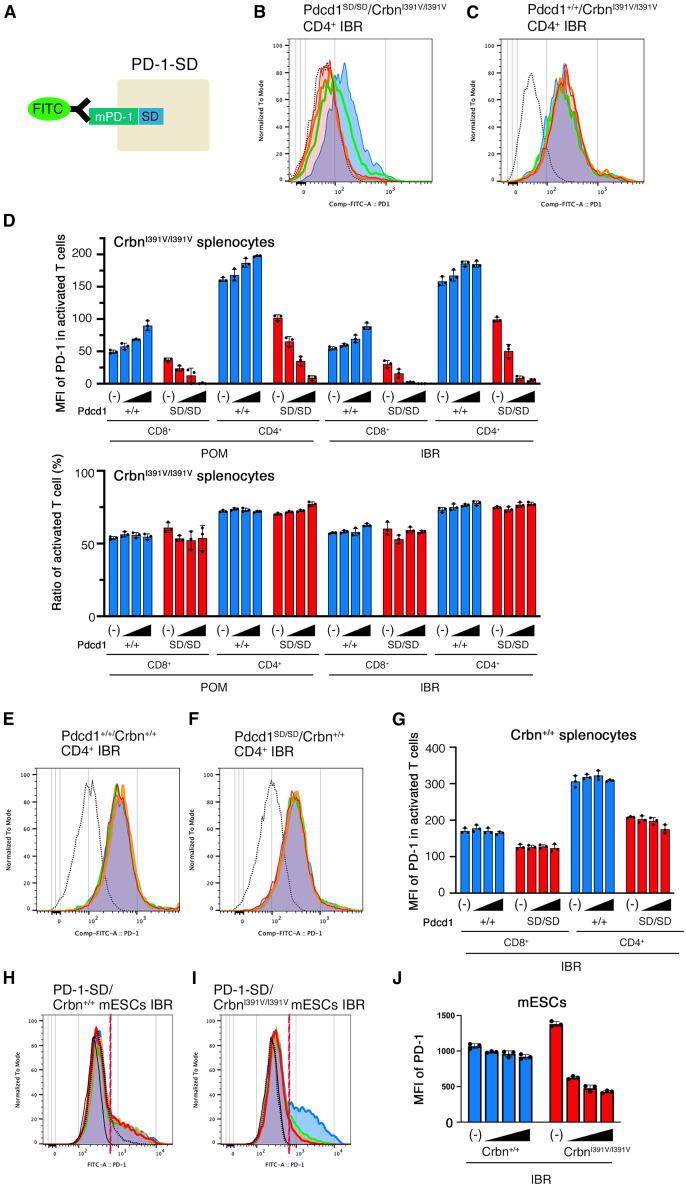
Degradation of endogenous PD-1 in mouse T cells by the addition of iberdomide after stimulation with concanavalin A (A) Schematic diagram of SD-tagged mouse endogenous PD-1 (mPD-1) on cell surface and detection by FITC-bound anti-mouse PD-1. (B and C) Typical examples of PD-1 expression on CD4^+^ T cells of *Pdcd1*^+/+^/*Crbn*^I391V/I391V^ (B) and *Pdcd1*^SD/SD^/*Crbn*^I391V/I391V^ (C) with iberdomide (IBR) after stimulation with concanavalin A analyzed by flow cytometry. The concentration of IBR is 0 (blue), 10^−3^ (green), 10^−2^ (orange), and 10^−1^ μM (red). The dotted lines show an isotype control without IBR. (D) The amount of PD-1 on CD4^+^ and CD8^+^ T cells of *Pdcd1*^+/+^/*Crbn*^I391V/I391V^ (+/+) and *Pdcd1*^SD/SD^/*Crbn*^I391V/I391V^ (SD/SD) (top) and ratio of activated T cells (bottom) with various concentrations of pomalidomide (POM) and IBR. (E and F) Typical examples of PD-1 expression on CD4^+^ T cells of *Pdcd1*^+/+^/*Crbn*^+/+^ (E) and *Pdcd1*^SD/SD^/*Crbn*^+/+^ (F) with IBR analyzed by flow cytometry. The concentration of IBR is 0 (blue), 10^−3^ (green), 10^−2^ (orange) and 10^−1^ μM (red). The dotted lines show an isotype control without IBR. (G) The amount of PD-1 on CD4^+^ and CD8^+^ T cells of *Pdcd1*^+/+^/*Crbn*^+/+^ (+/+) and *Pdcd1*^SD/SD^/*Crbn*^+/+^ (SD/SD) with various concentrations of IBR. (H and I) Typical examples of PD-1 expressions on exogenous PD-1-SD-expressed *Crbn*^+/+^ mESCs (H) and that on *Crbn*^I391V/I391V^ mESC (I) with various concentrations of IBR analyzed by flow cytometry. The concentration of IBR is 0 (blue), 10^−3^ (green), 10^−2^ (orange), and 10^−1^ μM (red). The black lines show isotype control without IBR. The dotted line in H shows the result of 10^−1^ μM on *Crbn*^I391V/I391V^ mESC in I for comparison. The dotted line in I shows the result without transfection. Red lines show the boundary between positive and negative. (J) The amount of PD-1 on PD-1-positive mESCs of *Crbn*^+/+^ and *Crbn*^I391V/I391^ with various concentrations of IBR. (B–J) IBR or solvent (−) was added to cultured splenocytes (B–G) or mESCs (H–J). The triangles indicate the concentration of IBR. The concentration is 0.1 μM in the rightmost bar in each group, and the left neighbor is a step dilution of one-tenth. Intensities of anti-PD-1-FITC on CD4^+^ and CD8^+^ T cells were measured by flow cytometry, and the geometric means of fluorescence intensities (MFI) were calculated. The MFI of the isotype control of anti-PD-1-FITC was subtracted from each MFI of anti-PD-1-FITC. The dots indicate the results of the experiment for each of the three samples from different individuals. Data are represented as mean ± standard deviation.

Before knock-in of SD into the downstream of endogenous PD-1 in mice, the linker sequence linking mouse PD-1 to SD was then examined in Jurkat cells, since our previous results with SMASh tag showed that the leakage and degradation efficiency of PD-1 depended on the structure of the linker between PD-1 and degron tag (Figure S4A). We used human Jurkat cells as it was difficult to accurately assess exogenous PD-1 expression, as EL4 cells originally expressed mouse PD-1. PD-1 expression was significantly reduced by IBR with a linker (Figure S4B). The SD-tagged PD-1 expressed by these constructs without IBR was higher than that expressed by the no-tag construct, suggesting that PD-1-SD leakage was very low. Therefore, the simplest “no linker” construct was adopted for *in vivo* protein knockdown in mice. Considering that fluorescent proteins affect the degradation efficiency of PD-1,^31^ we decided to knock in without a fluorescent protein as a marker. SD was inserted downstream of the endogenous *Pdcd1* gene (the gene encoding PD-1) in *Crbn*^I391V/I391V^ mice using genome editing (Figures 6A and S5), and double homozygous (*Pdcd*1^SD/SD^/*Crbn*^I391V/I391V^) mice were produced by intercrossing.

*Pdcd*1^SD/SD^/*Crbn*^I391V/I391V^ spleen-derived T cells were stimulated *in vitro* with concanavalin A (Con A) followed by treatment with thalidomide analogs to examine PD-1 expression (Figures 6B–6D and S6A–S6C). PD-1 was barely observed in both CD8^+^ and CD4^+^ cells 24 h after the addition of 1 μM POM or 0.1–1 μM IBR, therefore, the addition of 0.1 μM IBR appeared to be the most suitable condition for PD-1 knockdown in T cells. PD-1 expression in Con A-stimulated *Pdcd1*^SD/SD^/*Crbn*^I391V/I391V^- T cells was lower than that in *Pdcd1*^+/+^/*Crbn*^I391V/I391V^ cells, even without IBR treatment, which was considered leakage. The percentage of activated T cells characterized by FSC^high^ was not affected by the presence or absence of POM or IBR (Figure 6D).

Because EGFP-SD was degraded in *Crbn*^+/+^ mESCs in an IBR concentration-dependent manner, we investigated whether PD-1-SD could also be degraded in *Pdcd1*^SD/SD^/*Crbn*^+/+^ mouse splenocytes by adding IBR *in vitro*. Unexpectedly, PD-1 was barely degraded in *Pdcd1*^SD/SD^/*Crbn*^+/+^ T cells (Figures 6E–6G and S6D). To determine whether the same phenomenon occurs in non-T cells, exogenous PD-1 degradation was examined in mESCs transfected with *PD-1-SD* expression vectors (Figures 6H–6J and S6E). PD-1 was barely degraded in *Crbn*^+/+^ mESCs, whereas it was degraded in *Crbn*^I391V/I391V^ mESCs (Figure 6J). Moreover, significant leakage was observed in *Crbn*^+/+^ mESCs transfected with PD-1-SD expression vectors compared to *Crbn*^I391V/I391V^ mESCs. We hypothesized that the degradation efficiency of membrane-localized proteins might be reduced in mouse-type CRBNs. Therefore, we expressed EGFP-SDs with membrane-localized peptides attached to their N-termini in mESCs and added IBR. The degradation efficiency of EGFP-SD with membrane-localization peptide^32^ (MLS-EGFP-SD) in *Crbn*^+/+^ ESCs was lower than that of EGFP-SD, unlike *Crbn*^I391V/I391V^ mESCs (Figure S7), although the degradation efficiency was higher than that of PD-1-SD in *Crbn*^+/+^ ESCs. This result may partially explain the low efficiency of PD-1-SD degradation in *Crbn*^+/+^ cells. Therefore, *Pdcd1*^SD/SD^/*Crbn*^I391V/I391V^ mice were used for further analyses.

### Degradation of mouse endogenous PD-1 *in vivo*

**Figure 7:**
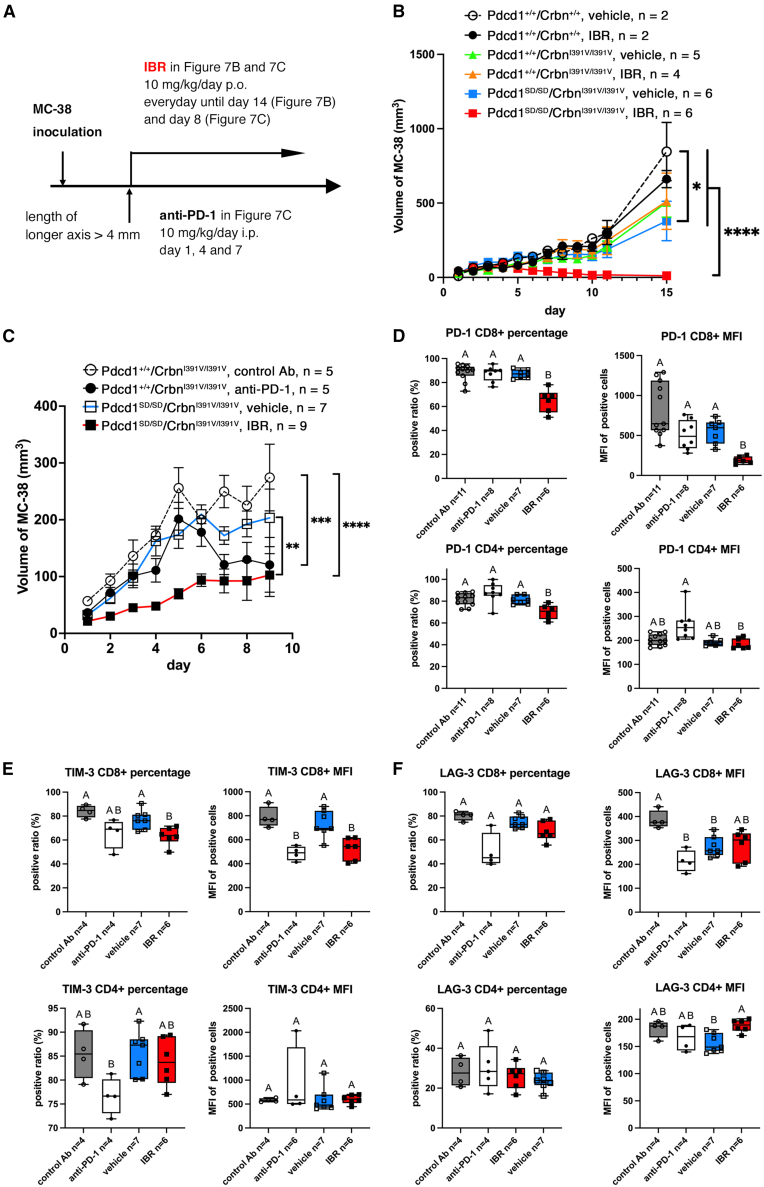
Administration of iberdomide repressed the proliferation of MC-38 adenocarcinoma cells in *Pdcd1*^SD/SD^/*Crbn*^I391V/I391V^ mice (A) Schedule for the experiment of MC-38 inoculation and administration of iberdomide (IBR) or anti-PD-1 antibody. The size of MC-38 cell clumps was measured daily by electronic calipers starting 1 day after inoculation; the first day of IBR and anti-PD-1 administration was the day when the length of the long axis of the MC-38 clump reached 4 mm. (B) Effects of iberdomide (IBR) on the growth of MC-38. The numbers of mice are shown in the figure. Data are represented as mean ± standard error (SEM). The same experiment was repeated twice to confirm reproducibility. *p*-values were determined by two-way ANOVA multiple comparisons. ∗∗∗∗*p* < 0.0001 and ∗*p* = 0.0267. (C) Effects of anti-PD-1 antibodies in *Pdcd1*^+/+^/*Crbn*^I391V/I391^ mice and IBR in *Pdcd1*^SD/SD^/*Crbn*^I391V/I391^ mice on MC-38 proliferation. The numbers of mice are shown in the figure. Data are represented as mean ± SEM. The same experiment was repeated twice to confirm reproducibility. *p*-values were determined by two-way ANOVA multiple comparisons. ∗∗∗∗*p* < 0.0001, ∗∗∗*p* = 0.0005, ∗∗*p* = 0.0067. (D–F) *Pdcd1*^+/+^/*Crbn*^I391V/I391^ mice and *Pdcd1*^KI/KI^/*Crbn*^I391V/I391^ mice were used for antibody treatment (control Ab and anti-PD-1) and IBR treatment (vehicle and IBR), respectively. Ratio of PD-1 positive cells and mean fluorescence intensity (MFI) of PD-1 (D), TIM-3 (E), and LAG-3 (F) on CD8^+^ and CD4^+^ T cells isolated from tumor microenvironments 9 days after the initiation of treatment are shown. Fluorescence intensity of each molecule was measured by flow cytometry, and the geometric MIFs of marker-positive cells were calculated. The numbers of mice are shown in the figure. The data in two experiments were compiled. *p*-values were determined by the Brown-Forsythe and Welch ANOVA test. Different letters indicate statistically significant differences between groups (*p* < 0.05). Boxplots: hinges, 25^th^ and 75^th^ percentiles; middle line, median; whiskers, minimum to maximum value.

The MC-38 adenocarcinoma cell line was transplanted into wild-type (*Pdcd1*^+/+^/*Crbn*^+/+^), *Pdcd1*^+/+^/*Crbn*^I391V/I391V^, and *Pdcd1*^SD/SD^/*Crbn*^I391V/I391V^ mice to examine the effects of the oral administration of IBR on endogenous PD-1 degradation *in vivo* (Figure 7A). The growth of MC-38 cells was suppressed in *Pdcd1*^SD/SD^/*Crbn*^I391V/I391V^ mice treated with IBR compared to that in all other groups, including untreated *Pdcd1*^SD/SD^/*Crbn*^I391V/I391V^ mice (Figures 7B, S8A, and S8B). Additionally, MC-38 cell growth in *Pdcd1*^SD/SD^/*Crbn*^I391V/I391V^ mice without IBR was significantly reduced compared to that in *Pdcd1*^+/+^/*Crbn*^+/+^ mice without IBR, probably because of unexpected PD-1 degradation.

Next, the effects of SD-induced PD-1 reduction were compared with those of anti-PD-1 antibody treatment *in vivo*. A comparison of *Pdcd1*^+/+^/*Crbn*^I391V/I391V^ mice treated with the anti-PD-1 antibody and *Pdcd1*^SD/SD^/*Crbn*^I391V/I391V^ mice treated with IBR showed that MC-38 cell proliferation was suppressed after a transient increase with anti-PD-1 antibody treatment, whereas IBR treatment suppressed proliferation for the entire period (Figure 7C). CD3^+^ T cells infiltrating the MC-38 microenvironment were analyzed on day 9 of treatment before the MC-38 tumor masses had shrunk with treatment and no longer provided sufficient T cells for FACS analyses. The frequency of infiltrating T cells was similar among the experimental groups (Figures S8C and S8D).

Subsequently, we analyzed the proportion of exhaustion and activation marker-positive cells in each experimental group, as well as the expression levels of each marker in the marker-positive cells. IBR-treated *Pdcd1*^SD/SD^/*Crbn*^I391V/I391V^ T cells showed a significant reduction of the PD-1^+^ cell ratio in both CD8^+^ and CD4^+^ T cells and of PD-1 expression level in PD-1^+^ CD8^+^ T cells compared to untreated *Pdcd1*^SD/SD^/*Crbn*^I391V/I391V^ T cells, even though oral administration was completed one day before the experiment (much longer than the T_1/2_ of IBR *in vivo*^33^) (Figures 7D, S8E, and S8F), whereas, neither PD-1^+^ cell ratio nor PD-1 expression level in *Pdcd1*^+/+^/*Crbn*^I391V/I391V^ treated with the anti-PD-1 antibody were different from the untreated group (Figure 7D).

In IBR-treated *Pdcd1*^SD/SD^/*Crbn*^I391V/I391V^ CD8^+^ T cells, both the TIM-3^+^ cell ratio and TIM-3 expression level in TIM-3^+^ cells were also decreased compared to the untreated group, whereas other exhaustion markers, LAG-3 and TIGIT, remained comparable (Figures 7E, 7F, and S8G). CX3CR1, which is expressed in the activated T cells, was comparable between IBR-treated and untreated groups (Figure S8H). In contrast, the antibody treatment of *Pdcd1*^+/+^/*Crbn*^I391V/I391V^ CD8^+^ T cells resulted in a reduction of TIM-3, LAG-3, and TIGIT in expression level whereas CX3CR1^+^ cell ratio was reduced compared to the untreated group. In IBR-treated *Pdcd1*^SD/SD^/*Crbn*^I391V/I391V^ CD4^+^ T cells, both the ratio of marker-positive cells and the expression level of markers were comparable to those in the untreated groups, except that LAG-3 expression levels were higher than those in the untreated group.

*In vivo* proliferation of another tumor cell line, GL-261 neuroblastoma, was also examined to confirm the effect of PD-1 reduction other than the MC-38 adenocarcinoma cell line.^34^ Proliferation of GL-261 was also suppressed in the *Pdcd1*^SD/SD^/*Crbn*^I391V/I391V^ mice with IBR oral administration compared to non-treated *Pdcd1*^SD/SD^/*Crbn*^I391V/I391V^ and IBR-treated *Pdcd1*^+/+^/*Crbn*^I391V/I391V^ mice (Figure S9), suggesting the growth of highly immunogenic tumor cell lines might be inhibited in a similar method.

One of the most commonly used degron systems is the use of dTAG and dTAG-13; however, it cannot be used for the degradation of proteins in the brain because dTAG-13 cannot cross the blood-brain barrier (BBB).^35^ Although the growth of GL-261 in the brain was inhibited, it is likely that the BBB was destroyed in cancer growth experiments in which GL-261 was transplanted.^34^ Therefore, we conducted an experiment in which IBR was administered without the transplantation of brain tumor cell lines as a model for protein degradation experiments in the brain under normal conditions. Since there are no reports on whether IBR crosses the BBB, we orally administered IBR to *Pdcd1*^SD/SD^/*Crbn*^I391V/I391V^ mice and found no changes in PD-1 expression in the brain (Figure S10A). Therefore, we orally administered POM, which passes through the BBB.^36^ In *Pdcd1*^SD/SD^/*Crbn*^I391V/I391V^ mice treated with 20 mg/kg POM, PD-1 was reduced to half the amount after 24 h compared to mice treated with vehicle (Figures S10B–S10D). Thus, this degron system can be used in the brain.

The important functions of PD-1 are to suppress autoimmunity, and depletion or suppression of PD-1 leads to autoimmune diseases.^37^ To determine the immunological effect by *Pdcd1*^SD/SD^/*Crbn*^I391V/I391V^, entire organs of each individuals which included heart, lungs, liver, kidneys, pancreas, spleen, adrenal glands, small and large intestine, bone marrow, joints of lower extremities, salivary glands (submandibular), lacrimal glands and dorsal skin from 12-month old mice were histopathologically analyzed by skilled pathologist, then resulted into the definition of no significant organ injury in the *Pdcd1*^+/+^/*Crbn*^I391V/I391V^ mice as well as in *Pdcd1*^SD/SD^/*Crbn*^I391V/I391V^ mice with or without MC-38 inoculation and IBR treatment (Figures S11 and S12). These results suggested that there was minimal leakage of PD-1 degradation *in vivo*.

## Discussion

In this study, we evaluated the effectiveness of the degron system using SD and IBR in mouse cells and whole body. Initially, we demonstrated their effect on EGFP degradation in mouse cell lines (Figures 2, 3, 4, and 5). Second, we observed that PD-1 degradation did not occur in wild-type mouse cells but only in Crbn^I391V/I391V^ mouse cells (Figure 6). This led us to further discuss the challenges associated with membrane protein degradation. Finally, we successfully achieved PD-1 degradation *in vivo* (Figure 7).

There are four key factors for assessing the degradation ability of the degron tag: (1) degradation efficiency, which allows protein degradation with fewer drugs; (2) protein reduction rate at the plateau; (3) time to reach the plateau; and (4) leakage in the absence of drugs. (1) In the mouse wild-type cell lines, IBRs of approximately 10^−2^ μM to 10^−3^ μM, typical doses of many chemicals used in cells and animal studies, suppressed stably expressed exogenous SD-EGFP and EGFP-SD to 20% and 10%, respectively. However, about 10- to 100-fold IBRs were required to reduce protein levels to the same extent as in human cell lines (Figure 3), therefore, CRBN-humanized mouse cells and mice are generally suitable for CRBN-based chemical knockdown experiments with most efficient decrease of protein levels, corresponding to the previous studies.^21^^,^^22^^,^^23^ However, it suggests that it is easier in wild-type mouse cells and mice to reduce the protein to desired levels, rather than complete knockdown. In human cells, on the other hand, it is expected to be difficult to control the level of protein degradation because of their very sensitive response to drugs. (2) The expression of EGFP at the lowest level in wild-type mouse cell lines was comparable to that in human cell lines, although there were differences in the IBR concentrations required to achieve the lowest level (Figure 3). In wild-type mouse cells, the degradation of SD-tagged EGFP was CRBN-dependent, as in human cells (Figure 2J), despite the structural differences between mouse and human CRBNs (Figure 1B). These results suggested that the degradation of SD-tagged EGFP occurs via the same mechanism in mice as in human cells. (3) In the mouse wild-type cell lines, the time to halve the amount of EGFP-SD after the addition of IBR was approximately 20–40 min, and the time to reduce it to 10% was approximately 3 h, independent of the IBR concentration in the test (Figure 4). These results suggest that this system is suitable for the analysis of phenomena such as DNA replication, the cell cycle, signal transduction, and epigenetic regulation in mouse cells, where changes occur within a few hours. (4) In the absence of IBRs, degradation leakage was greater in mouse cells than in human cells (Figure 3). In particular, the leakage in mESCs and rESCs was much greater than that in hESCs, but not as much in NIH/3T3 and EL4 cells, suggesting that it might depend on the cell type. In ESCs, NIH/3T3, and HEK293 cells, SD-EGFP (N-terminal SD) was less expressed than EGFP-SD (C-terminal SD) under IBR-free conditions, suggesting a higher leakage of degradation of the N-terminal degron tag.

Only SD was able to efficiently degrade EGFP even in the wild-type mouse cell lines. IKZF1/3 and several neo-substrates for CRBN were degraded in human cells but not in mouse cells, most likely because of the different amino acids in CRBN. However, the combination of SD and IBR or MZG degraded target proteins in both mouse and human cells. This may be because the addition of IBR and MZG, which can bring the loop structure of CRBN closer to the SD, allows the SD structure to bind to both human and mouse CRBN despite structural discrepancies (Figure 5C). The combination of the SD and IBR or MZG specifically and efficiently degraded the target protein, while IKZF and ZFP91 tags degraded EGFP by approximately 10% (Figure 5). In addition, the specificity of IBRs was examined using a proteome assay, which revealed that the addition of IBRs to wild-type mESCs reduced the expression of 17 proteins. These 17 proteins are suspected to be novel substrates (data not shown). Of these, 11 candidates whose cDNAs were successfully cloned bound to EGFP and were exogenously expressed in mESCs with the addition of IBR; however, none of them were downregulated by IBR and were therefore considered false positives. Moreover, general thalidomide analogs and CRBN have no neo-substrates in mouse cell lines.^25^ Recent research shows that very few proteins in the Ba/F3 mouse pro-B cell line were reduced by the addition of pomalidomide.^38^ These facts suggest that thalidomide analogs might have a few off-targets in wild-type mouse cells; therefore, the combination of SD and IBR might be a suitable protein degradation system in wild-type mouse cells, if the target protein could be degraded.

Surprisingly, in the human Jurkat cell line, the PD-1-IKZF and PD-1-Sall4 were not efficiently degraded by POM, and only PD-1-SD was degraded (Figure 1). Furthermore, PD-1-SD was barely degraded by IBR in *Pdcd1*^SD/SD^/*Crbn*^+/+^ mouse splenocytes and wild-type mESCs (Figure 6), although EGFP-SD was efficiently degraded by IBR in wild-type mESCs (Figure 3). The difference in the degradation activity of different target proteins may be due to differences in the degradation pathways, which are influenced by the distinct subcellular localization of EGFP (mainly in the cytosol) and PD-1 (membrane). It remains to be determined which proteins this degron system is applicable to and which degradation pathways are used. Intracellular organelles that can or cannot use proteolytic tags and drugs have been analyzed based on their subcellular localization^39^; however, membrane proteins have not yet been described. Some methods to degrade membrane proteins, such as Lytac, have been developed mainly for therapeutic purpose, however, it requires finding baits specific for the target protein and it is not convenient for the usage for basic biology.^40^ In this study, we succeeded in the efficient degradation of PD-1 by binding an SD to the intracellular terminus of PD-1. PD-1 degradation was originally regulated by ubiquitination by FBXO38.^41^ Moreover, the turnover time of PD-1 is approximately 3 h when activated.^41^ Therefore, this system may be applicable to membrane proteins whose expression is regulated by ubiquitination and whose turnover is rapid.

In *Pdcd1*^SD/SD^/*Crbn*^I391V/I391V^ mouse T cells, SD bound to endogenous PD-1, and IBR efficiently reduced PD-1 in both CD4^+^ and CD8^+^ T cells *in vitro*. PD-1 was mostly removed in *Pdcd1*^SD/SD^/*Crbn*^I391V/I391V^ T cells at 10^−2^ μM and 10^−3^ μM IBR (Figure 6D). In the microenvironment of MC-38 in *Pdcd1*^SD/SD^/*Crbn*^I391V/I391V^ mice treated with IBR, the proportion of PD-1^+^ cells among both CD4^+^ and CD8^+^ T cells, as well as the PD-1 expression level in CD8^+^ T cells, was significantly lower compared to the untreated group, even 1 day after the last administration of IBR (Figure 7D). The pharmacokinetics in mouse administered IBR by oral administration in BALB/c mice showed the concentration in serum peaks at 0.14 h and the half-life (T_1/2_) is 0.9 h after the administration.^33^ Moreover, based on the result of oral administration in humans, the concentration in serum peaks 3 h after administration, both single and multiple once-daily doses of IBR.^42^ When multiple doses are administered in humans, the serum concentration of IBR doubles compared to a single dose, and even after 24 h, IBR is present at about 20% of the maximum concentration, which may be sufficient to keep PD-1 levels low even in the mouse model. Because lymphocyte differentiation is suppressed in *Crbn*^I391V/I391V^ mice,^25^ the number of T cells may decrease, and their ability to eliminate tumors may be reduced by injecting IBR for a long time. However, the number of T cells infiltrating the microenvironment did not differ between the IBR- and vehicle-treated groups (Figure S8D), suggesting that the IBR treatment conditions used in this study had little inhibitory effect on cancer immunity.

Degradation of PD-1 appears to act more rapidly than antibody treatment, as suggested by the suppression of MC-38 growth in Figure 7C. Upon the knockdown of PD-1 by IBR treatment, not only PD-1 positive population and expression level but also those of TIM-3 were suppressed in CD8^+^ T cells. In contrast, other exhaustion markers such as LAG-3 (which is expressed in the early phase of T cell activation and is mainly involved in the activation-restricted pathway of T cells) and TIGIT (which is expressed in the final exhaustion phase)^43^ were not suppressed by knockdown, however, those were suppressed by anti-PD-1 antibody treatment. The expression levels of LAG-3 and TIGIT by PD-1 knockdown, similar to those in the untreated group, are likely due to stronger PD-1 functional suppression compared to antibody blockade. This stronger inhibition may have induced a compensatory upregulation of these molecules. Therefore, to effectively suppress T cell exhaustion through PD-1 knockdown, further investigation is warranted regarding the timing and extent of knockdown. Nevertheless, the growth of MC-38 was inhibited in knockdown of PD-1 by IBR treatment. Although synergistic tumor suppression via PD-1 and LAG-3 inhibition has been reported,^44^^,^^45^ PD-1 is considered to play a primary role in the suppression and elimination of MC-38 cells. Since IBR activates natural killer (NK) cells via both Zap-70 and the CRBN-dependent pathway,^46^ it is possible that MC-38 elimination was caused by activated NK cells rather than PD-1 reduction. However, MC-38 proliferation in *Pdcd1*^+/+^/*Crbn*^I391V/391V^ mice treated with IBR and with vehicle was comparable, suggesting that PD-1 suppression, not IBR treatment, might be the cause of MC-38 elimination in this study. These findings may be applicable to prevent CAR-T cell exhaustion by regulating PD-1 expression levels.

At present, SD is considered an excellent degron system in mice, balancing protein degradation efficiency with the cost of reagents used for degradation. There are several IMiD-based tags available for use in mouse cells.^27^^,^^38^ Although a simple comparison is difficult because the cell lines are not the same as in the present study, the degradation efficiency of EGFP by the degron system with DCD23 tag and IBR in mouse cell lines is lower than that of the SD system.^27^ Recently, improved degron tags and thalidomide analogs have been developed that can be used in mouse cells using a phage-assisted continuous evolution platform.^38^ The new combination SD56 tag and a thalidomide analog, PT-179, could potentially be used in mice *in vivo* with almost the same efficiency and cost as the degron system using SD and IBR. In addition to thalidomide analogs, the degron system using dTAG-13 is well known. Small molecules, such as dTAG-13, are not delivered across the blood-brain barrier into the brain; however, POM passes through the blood-brain barrier^36^ and PD-1 in the brains of *Pdcd1*^SD/SD^/*Crbn*^I391V/I391V^ mice was reduced compared to that in control mice (Figure S10), although the conditions of more efficient degradation should be examined. The SD degron system worked better *in vivo* than our previously used SMASh tag^14^: the SMASh tag showed greater leakage and a smaller reduction in the target protein when asunaprevir (ASV) or grazoprevir (GRV) was added, whereas SD showed less leakage and a greater rate of protein reduction. However, further studies on substrate specificity in mice are required before the SD-IBR degron system can be used. In addition, the effects of the degradation of neo-substrates, such as IKZF1/3, in CRBN human-type mice need to be considered. To solve this problem, it is necessary to search for and develop more SD-specific agents, or agents that can degrade SD at such low volumes that other neo-substrates are not degraded.

In summary, the degron system offers a less toxic and faster-acting alternative to conventional methods, such as doxycycline. It is thought that endogenous proteins can be degraded efficiently in mouse cells and the mouse body by expressing target protein-linker-SD vectors and examining the conditions of CRBN (human or mouse type) that can be degraded in mouse cells before being applied to mice. This approach could enhance studies in fields such as immunology and neurobiology by enabling rapid protein loss analysis, identifying optimal intervention times for diseases with known causative proteins, and allowing precise control of protein expression levels. These advancements can lead to impactful new research directions with broad international significance.

### Limitations of the study

The inability to accurately measure PD-1 reduction *in vivo* was a major technical limitation of this study. The lack of accurate measurements makes it difficult to fully understand the dynamics of PD-1 expression and how it changes over time in response to treatment. Without these data, it would be difficult to draw clear conclusions regarding effective knockdown. One of the advantages of using SD in mice is that it does not require genetic modifications other than the protein of interest. However, as this study has shown, some proteins require the use of mice transfected with human CRBN. While demonstrating the potential of the super-degron system in mouse models, the variability in response across different cell types and conditions indicates the need for further studies, including the elucidation of proteolytic pathways, to generalize the results.

## Resource availability

### Lead contact

Requests for further information and resources should be directed to and will be fulfilled by the lead contact Chie Naruse (naruse.chie.6r@kyoto-u.ac.jp).

### Materials availability

All materials generated in this study are available from the lead contact upon reasonable request.

### Data and code availability

• Data: Data are available at https://data.mendeley.com/datasets/md324cfjv3/2.
• Code: No custom code was used in this study.
• Other: All information is available from the lead contact upon request.

## Acknowledgments

This work was supported by the 10.13039/501100005683Kyoto University Live Imaging Center. Rat ESC was kindly provided by Dr Toshihiro Kobayashi (The University of Tokyo). This work is supported by the Grant-in-Aid for Scientific Research (B) JP20H03171 (C.N.), Grant-in-Aid for Challenging Research (Exploratory) JP21K19188 (C.N.) and AdAMS JP22H04922 (C.N.) from 10.13039/501100001691Japan Society for the Promotion of Science, Japan and The 10.13039/100016926Kyoto University Foundation, Japan (C.N.). We thank the excellent care of mice by the staff of the Institute of Laboratory Animals, Graduate School of Medicine, Kyoto University. We would like to thank Editage (www.editage.jp) for English editing.

## Author contributions

Conceptualization, C.N., O.I, and M.A.; investigation, C.N., O.I., M.O., H.I., T.M., X.P., T.M., Y.S., and Y.S.; writing–original draft, C.N., M.O., H.I., T.M., and Y.S.; writing – review and editing, F.S. and M.A.; project administration, C.N. and M.A.; supervision, C.N. and M.A.; funding acquisition, C.N.

## Declaration of interests

The authors declare no competing interests.

## Declaration of generative AI and AI-assisted technologies in the writing process

During the preparation of this work, the authors used ChatGPT in order to improve the clarity and readability of the English text. After using this tool, the authors reviewed and edited the content as needed and take full responsibility for the content of the publication.

## STAR★Methods

### Key resources table

**Table:** 

REAGENT or RESOURCE	SOURCE	IDENTIFIER
**Antibodies**		

Mouse monoclonal anti-CRBN	Abcam	Cat# Ab244223
Horse polyclonal anti-mouse IgG (H+L)	Vector Laboratories	Cat# BA-2000, RRID: AB_2313581
Rabbit polyclonal anti-beta-Actin	Bioss	Cat# bs-0061R, RRID: AB_10855480
Goat polyclonal anti-Rabbit IgG H&L (HRP)	Abcam	Cat# ab6721, RRID: AB_955447
APC/Cyanine7 rat monoclonal anti-mouse CD3	BioLegend	Cat# 100222, RRID: AB_2242784
Pacific Blue rat monoclonal anti-mouse CD4	BioLegend	Cat# 100428, RRID: AB_493647
Alexa Fluor rat monoclonal anti-mouse CD8a	BioLegend	Cat# 100730, RRID: AB_493703
FICT rat monoclonal anti-mouse CD279 (PD-1)	BioLegend	Cat# 135214, RRID: AB_10680238
PE/Cyanine7 rat monoclonal anti-mouse CD366 (Tim-3)	BioLegend	Cat# 119715, RRID: AB_2571932
PerCP/Cyanine5.5 rat monoclonal anti-mouse CD223 (LAG3)	BioLegend	Cat# 125211, RRID: AB_2561516
APC mouse monoclonal anti-mouse CX3CR1	BioLegend	Cat# 149007, RRID: AB_2564491
PE mouse monoclonal anti-mouse TIGIT (Vstm3)	BioLegend	Cat# 142103, RRID: AB_10895760
Rat monoclonal Anti-mouse PD-1 (CD279)-InVivo clone RMP1-14	Selleck	Cat# A2122, RRID: AB_3644244
Rat IgG2a isotype control-InVivo	Selleck	Cat# A2123, RRID: AB_3644245
Rabbit monoclonal anti-mouse PD-1	Abcam	Cat# ab214421, RRID: AB__2941806
Rabbit polyclonal anti-beta-actin	Bioss	Cat# bs-0061R, RRID: AB_10855480
Goat polyclonal anti-rabbit IgG HRP conjugated	Abcam	Cat# ab6721, RRID: AB_955447

**Chemicals, peptides, and recombinant proteins**		

Streptavidin poly-HRP	Pierce	Cat# 21140
Zombie Aqua Fixable Viability Kit	BioLegend	Cat# 423102
SYTOX Red dead cell stain, for 633 or 635 nm excitation	Thermo Fisher Scientific	S34859
pomalidomide	Selleck	Cat# S1567
iberdomide	Selleck	Cat# S8760
avadomide	WAKO	Cat# NCEHY-100507-5
mezigdomide	Selleck	Cat# S8975
concanavalin A	Sigma	Cat# C5275-5MG

**Experimental models: Cell lines**		

Human: Jurkat cells	N/A	N/A
Mouse: B6J-S1 ES cells	Tanimoto et al.^24^	N/A
Mouse embryonic fibroblasts	Naruse et al.^14^	N/A
Rat: ES cells	Hirabayashi et al.^45^	N/A
Human: KhES-1 ES cells	Suemori et al.^46^	N/A
Mouse: EL4	Riken BRC	Cat# RCB1641
Human: AAVpro293T	TakaraBio	Cat# 632273
Mouse: NIH/3T3	Riken BRC	Cat# RCB2767
Mouse: MC-38	Kerafast, Inc	Cat# ENH-204-FP
Mouse: GL-261	DSMZ Leibniz Institute	Cat# ACC802

**Experimental models: Organisms/strains**		

Mouse: *Crbn*^I391V/I391V^: B6-*Crbn*^tm2I391V^	This paper	N/A
Mouse: *Pdcd1*^SD/SD^: B6-*Pdcd1*^tm2SD^	This paper	N/A
Mouse: *Pdcd1*^SD/SD^/*Crbn*^I391V/I391V^ : B6- *Pdcd1*^tm2SD^/*Crbn*^tm2I391V^	This paper	N/A

**Oligonucleotides**		

Primers, gRNAs, single strand oligoDNAs are shown in Table S6	N/A	N/A

**Recombinant DNA**		

pCAGGS	RIKEN BRC	Cat# RDB08938
pCAGp-PD1-mC-IKZF3s-P2A-Neo-pA	This paper	Table S7
pCAGp-PD1-mC-IKZF3L-P2A-Neo-pA	This paper	Table S7
pCAGp-PD1-mC-S4D-P2A-Neo-pA	This paper	Table S7
pCAGp-PD1-mC-SD-P2A-Neo-pA	This paper	Table S7
pCAGp-SD-EGFP-2A-mCherry-2A-Neo-pA	This paper	Table S7
pCAGp-EGFP-SD-2A-mCherry-2a-Neo-pA	This paper	Table S7
pCAGp-EGFP-2A-mCherry-2A-Neo-pA	This paper	Table S7
pCAGp-mCherry-2A-Neo-pA	This paper	Table S7
pCAGp-hyPBase-pA	This paper	Table S7
p(PB ITR)-CAGp-EGFP-SD-2A-mCherry-2a-Neo-pA	This paper	Table S7
p(PB ITR)-CAGp-SD-EGFP-2A-mCherry-2a-Neo-pA	This paper	Table S7
p(PB ITR)-CAGp-mCherry-2A-Neo-pA	This paper	Table S7
p(PB ITR)-CAGp-EGFP-IKZF-2A-mCherry-2a-Neo-pA	This paper	Table S7
p(PB ITR)-CAGp-EGFP-ZFP91-2A-mCherry-2a-Neo-pA	This paper	Table S7
pCAGp-PD-1-SD 1 SG	This paper	Table S7
pCAGp-PD-1-SD 2 hydrophil	This paper	Table S7
pCAGp-PD-1-SD 3 EA4K	This paper	Table S7
pCAGp-PD-1-SD 4 XP7	This paper	Table S7
pCAGp-PD-1-SD no linker	This paper	Table S7
pCAGp-PD-1 (no SD)	This paper	Table S7

**Software and algorithms**		

ImageJ	Schneider et al.^47^	https://imagej.net/software/imagej/
PyMOL	Schroedinger, Inc	https://www.pymol.org/
Prism	GraphPad Software	https://www.graphpad.com/company

**Deposited data**		

Supplemental figures and tables dataset	Mendeley Data	https://data.mendeley.com/datasets/md324cfjv3/2

### Experimental model and study participant details

#### Cell lines and cell culture

All cells were cultured at 37°C in a 5% CO_2_ atmosphere. The cell lines obtained are listed in Key resources table. The conditions of culture medium were as follows; Jurkat cell: RPMI-1640 (FUJIFILM Wako, Osaka, Japan) containing 10% Fetal Bovine Serum (FBS) (S-FBS-NL-015, Cosmo Bio Co., Ltd, Tokyo, Japan), 1% GlutaMAX supplement (Thermo Fisher Scientific, USA) and 100 U/mL of penicillin and 100 μg/mL streptomycin (Thermo Fisher Scientific). EL4 and NIH/3T3 cells: DMEM (low glucose) (FUJIFILM Wako) containing 10% FBS, 1% GlutaMAX, and penicillin-streptomycin. HEK293 cells: DMEM (FUJIFILM Wako) containing 10% FBS, 1% GlutaMAX supplement, 1% MEM non-essential amino acid solution (Thermo Fisher Scientific) and penicillin-streptomycin. Mouse ES cells: Knockout DMEM (Thermo Fisher Scientific) containing 15% Knockout Serum Replacement (Thermo Fisher Scientific), 1% Glutamax, MEM non-essential amino acids (Thermo Fisher Scientific), 1 mM sodium pyruvate (Thermo Fisher Scientific), penicillin-streptomycin, 10^4^ U/mL leukemia inhibitory factor (BioLegend), 300 μM CHIR99021 (GSK-3 inhibitor, Wako), and 100 μM PD0325901 (MEK inhibitor, Wako) on C3H-Neo embryonic fibroblasts treated with 10 μg/mL mitomycin C (Sigma-Aldrich) for 3 h on gelatin-coated plate. Rat ES cells: 1:1 mixture of DMEM/F12 (Thermo Fisher Scientific) and Neurobasal medium (Thermo Fisher Scientific) containing 1% B-27 serum free supplement (Thermo Fisher Scientific), 0.5% GlutaMAX (Thermo Fisher Scientific), penicillin-streptomycin, 10^4^ U/mL rat leukemia inhibitory factor (Sigma-Aldrich), 300 μM CHIR99021 (GSK-3 inhibitor, Wako), and 100 μM PD0325901 (MEK inhibitor, Wako) on C3H-Neo embryonic fibroblasts treated with 10 μg/mL mitomycin C on gelatin-coated plate. Human ES cells were cultured in StemFit AK02N medium (Reprocell, Yokohama, Japan) containing penicillin-streptomycin on an iMatrix511(Nippi)-coated plate. Y-27632 (10 μM, Selleck) was supplemented to the media for 24 hours upon plating. MC-38 cells: DMEM with 10% FBS, 1% GlutaMAX, MEM non-essential amino acids, 1 mM sodium pyruvate and penicillin-streptomycin. GL-261 cells: DMEM with 10% FBS, 1% GlutaMAX, and penicillin-streptomycin.

#### Mice

All experiments using mice were conducted in accordance with the Fundamental Guidelines for the Proper Conduct of Animal Experiments and Related Activities in Academic Research Institutions under the jurisdiction of the Ministry of Education, Culture, Sports, Science, and Technology of Japan and were approved by the Animal Experimentation Committee of the Faculty of Medicine, Kyoto University. The mice were maintained under specific pathogen-free conditions at the Institute of Laboratory Animals, Kyoto University Graduate School of Medicine. Mice were maintained at 24 ± 2°C and 60 ± 5% humidity under a 14-h light (07:00−21:00)/dark cycle. Mice were fed F-2 (Funabashi Farm, Chiba, Japan) and water. The experiments were performed in a nonblinded manner. For the inoculation of MC-38 cells, female mice were used to prevent skin injury during the experimental period due to fighting or skin inflammation. A combination of three drugs was intraperitoneally administrated for anesthesia: medetomidine (Domitor; Meiji Seika Pharma, Tokyo, Japan) at 0.3 mg/kg body weight, midazolam (Dormicum; Astellas, Tokyo, Japan) at 4 mg/kg body weight, and butorphanol (Betolfal; Meiji Seika Pharma, Tokyo, Japan) at 5 mg/kg body weight. The final volume was adjusted with physiological saline at a dose of 10 mL/kg. When euthanasia was necessary, skilled personnel performed the cervical dislocation or used carbon dioxide inhalation.

#### Production of CRBN-humanized and SD knocked in mice at C-terminal of endogenous PD-1

*Crbn*^I391V/I391V^ and *Pdcd1*^SD/SD^ mice were generated by genome editing using CRISPR/Cas9 and homologous recombination of single-stranded DNA to generate c1170a and a1171g mutations for *Crbn* and the insertion of SD, respectively. Target sequences for genome editing and single-stranded DNA sequences for homologous recombination are shown in Table S6. To generate genome-edited mice, C57BL/6 female mice (for *Crbn* mutation) or *Crbn*^I391V/I391V^ female mice (for *Pdcd1* mutation) were treated with 0.1 mL of HyperOVA (Kyudo, Tosu, Japan) 64 h before *in vitro* fertilization (IVF) and 5 U of hCG (ASKA Animal Health, Tokyo, Japan) 16 h before IVF. Epididymal tails were collected from C57BL/6 male mice (for *Crbn* mutation) or *Crbn*^I391V/I391V^ male mice (for *Pdcd1* mutation) euthanized by cervical dislocation, collected, and cultured in CARD HTF medium for 1h before *in vitro* fertilization (ARK Resource, Kumamoto, Japan). Oviducts were collected from female mice euthanized by cervical dislocation, extracted into HTF medium, and co-cultured with sperm. The fertilized eggs were washed and transferred into CARD KSOM medium (ARK Resource, Kumamoto, Japan) after culture for 5 h. One-cell embryos were placed into Opti-MEM including 400 ng/μl Alt-R HDR donor oligo (single strand DNA for homologous recombination of *Crbn* or *Pdcd1* in Table S6, Integrated DNA Technologies, IA, US) and gRNA (Table S6, IDT) - Cas9 HiFi v3 (IDT) complex, then placed between the 5 mm electrodes of NEPA21 Super electroporator (Nepagene, Ichikawa, Japan) and electroporated to introduce nucleic acids and proteins according to the manufacturer’s instructions (Poring 225 V, 1.5 msec, interval 50 msec x4, decay 10% +, Transfer 20 V. 50 msec, interval 50 msec x5, decay 40% +/-). The following day, 2-cell embryos were transferred to the oviducts of anesthetized pseudo-pregnant mice (SLC Japan, Hamamatsu, Japan), which were confirmed to have mated the morning of the day. Genotypes of the genome-edited mice were determined by PCR using specific primers (Figures S1 and S5; Table S6) and Sanger sequencing (Azenta, Kawasaki, Japan). The two genome-edited mouse lines were bred with C57BL/6J females to obtain heterozygous mutant *Crbn*^+/I391V^ mice. *Crbn*^+/I391V^ mice were bred to obtain *Crbn*^I391V/I391V^ mice. Because the two lines of genome-edited mice exhibited the same phenotype, only one line was used for this study. Double-mutant mice with a homozygous *SD* insertion of *Pdcd1* allele (*Pdcd1*^SD/SD^) and *Crbn*^I391V/I391V^ were generated by intercrossing *Pdcd*1^+/SD^/*Crbn*^I391V/I391V^ mice. *Pdcd*1^SD/SD^/*Crbn*^+/+^ mice were generated by backcrossing *Pdcd*1^SD/SD^/*Crbn*^I391V/I391V^ mice with C57BL/6 to produce *Pdcd*1^+/SD^/*Crbn*^+/I391V^ mice and then intercrossing these mice. Genotypes of *Crbn* in the offspring were determined by PCR using primers that included locked nucleic acids (LNA; Ajinomoto, Tokyo, Japan), as shown in Table S6. Genotypes of *Pdcd1* in the offspring were determined by PCR using the primers listed in Table S6.

#### Mouse embryonic stem cells with humanized CRBN and CRBN KO

*Crbn*^+/+^, *Crbn*^+/I391V^ and *Crbn*^I391V/I391V^ mESCs were produced from blastocysts obtained by crossing *Crbn*^+/I391V^ mice. Fertilized eggs were obtained as previously described in former paragraph. On day 3, embryos were transferred into ESC culture medium on a 24-well plastic plate. After the inner cell mass was increased and ESCs were formed, they were passaged onto feeder cells (mitomycin C-treated embryonic fibroblasts from E13.5, embryos of G418 transgenic C3H/He). For genotyping, ESCs with feeder cells were treated with Accutase (Nacalai, Kyoto, Japan), washed with medium, and cultured in 24-well plastic plates for 4 h to remove the fibroblast cells. Then, ESCs in supernatant were collected and lysed with PCR lysis buffer (10 mM Tris-HCl, 50 mM KCl, 0.45% NP-40, 0.45% Tween20) with 0.5 mg/mL proteinase K at 55°C overnight. Polymerase chain reaction (PCR) was performed to determine the genotypes of the ESCs using primers including LNA, as shown in Table S6. More than two clones of the same genotype were obtained, and two clones for each genotype were examined for protein degradation in the first experiment in Figure 1A. Because the phenotypes of the two clones were the same, one clone was examined in further studies. B6J-S1 ESCs derived from C57BL/6 wild-type mice^28^ were used as wild-type mESCs. B6J-S1 ESCs were transfected with the gRNA (Table S6) - Cas9 HiFi v3 complex using the Neon Transfection System at 1,400 V for 10 ms, three times (Thermo Fisher Scientific). ESC colonies from a single cell were picked, and the mutant clones were confirmed by Sanger sequencing of PCR products amplified with CRBN KO genotyping F and R primers (Table S6).

### Method details

#### Expression vector and transfection of the vectors into cell lines

The plasmid vectors and sequences are listed in key resources table and Table S7, respectively. DNA fragments encoding EGFP, linkers, P2A, T2A, mCherry, ITR for PiggyBac transposase, human AAVS, hyperactive piggyBac transposase^47^ and super-degron were synthesized by Fasmac Co., Ltd. (Kanagawa, Japan) or Vector Builder GmbH (Neu-Isenburg, Germany) and cloned into pCAGGS using the KOD-Plus-Mutagenesis Kit (TOYOBO, Osaka, Japan) and NEBuilder HiFi DNA assembly (NEB, MA US). The plasmid CAGp-hyPBase and the vector with the PB ITR were transfected to obtain stably expressing cells. Vectors other than human ESCs were transfected using the Neon Transfection System (Thermo Fisher Scientific) under the following conditions: Jurkat cells: 1,325 V, 10 ms, 3 times; mouse and rat ESCs: 1,400 V, 10 ms, 3 times; EL4 cells, 1,080 V, 50 ms; NIH/3T3 cells: 1,400 V, 20 ms, twice; HEK293 cells: 1,150 V 20 ms, twice. For hESCs, vectors were transfected using Lipofectamine Stem Reagents (Thermo Fisher Scentific) as described previously.^48^ The transfected cells were cultured with 1 mg/mL (besides ESCs) or 250 μg/mL (ESCs) of Geneticin Selective Antibiotic (Thermo Fisher Scientific) when selection was needed.

#### Fluorescence microscope observation

Cells expressing fluorescent proteins were observed under a fluorescence microscope (BZ-X710; Keyence). The experimental and control groups were photographed under the same conditions and the photographs were subjected to the same contrast processing and light-intensity adjustments using Adobe Photoshop 2024 (Adobe, CA, US).

#### Flow cytometry

Cells were harvested and stained with LIVE/DEAD fixable far red dead cell stain (Thermo Fisher Scientific, MA, US) or Zombi Aqua (BioLegend, CA, US) in FACS solution (2% FBS, 2 mM EDTA, 137 mM NaCl, 2.7 mM KCl, 10 mM Na_2_HPO_4_, 2 mM KH_2_PO_4_). The fluorescence intensities of the cells from the microenvironments were analyzed using a FACS Aria IIIu (BD Biosciences, NJ, US) and FlowJo v10 (BD Biosciences). When collecting microenvironments cells, mice were euthanized, MC-38 cell clumps were collected, washed gently with PBS (137 mM NaCl, 2.7 mM KCl, 10 mM Na_2_HPO_4_, 2 mM KH_2_PO_4_), and cut with a razor; cells were collected after incubation in Accutase at 37°C for 10 min and filtered through a 70 μm filter. The cells were then treated with ACT solution to remove erythrocytes, washed, and 1 × 10^6^ cells were used for flow cytometry.

#### Culture of splenocytes

Splenocytes were harvested after the mice were euthanized. Splenocytes were isolated in RPMI 1640 medium and collected using a 70 μm filter. After treatment with ACT solution to remove red blood cells, splenocyte culture was performed at a density of 2 × 10^6^ cells/mL in RPMI 1640 with 10% FBS, Glutamax and penicillin-streptomycin and incubated at 37°C in a 5% CO_2_ containing atmosphere. To activate T cells, 0.5 μg/mL concanavalin A was added to the medium for 3 days to activate T cells.

#### Inoculation of MC-38 colon adenocarcinoma into mice and IBR or anti-PD-1 antibody treatment

MC38 cells were passaged one day before inoculation, and 1 × 10^6^ cells per head were inoculated subcutaneously into anesthetized female mice at the age–3-4 months using syringes fitted with 23G needles (Terumo, Tokyo, Japan). IBR (10 mg/kg/day) was administered by intraperitoneal injection 4–5 days after inoculation with MC-38 cells, the time point at which the tumor size reached more than 45 mm. ASV and GRV were dissolved in DMSO to obtain 80 mg/mL and 40 mg/mL, respectively, and vortexed with nine times the volume of corn oil to form an emulsion; 50 μL of each solution was then administered intraperitoneally. The control group was administered DMSO adjusted in a similar manner. Anti-PD-1 antibody (10 mg/kg/day) and isotype control (10 mg/kg/day) were injected intraperitoneally on days 1, 4, and 7 (Figure 7A). Tumor size was measured daily using a digital caliper. The tumor volume was calculated using the following formula: volume = (length of longer axis) × (length of shorter axis)^2^ × 0.52.^49^^,^^50^ The endpoints of the cancer transplantation experiment were a tumor size of 2,000 mm^3^, weight loss of 20% or more from the start of the experiment, and debilitation.

#### GL-261 glioma orthotopic model

GL-261 cells were passaged one day before inoculation and resuspended in HBSS at a concentration of 1 × 10^7^ cells/mL. The mice were anesthetized by intraperitoneal injection of a combination of the three drugs described in the previous section and immobilized in a stereotactic frame (RWD Life Science, US). After a midline scalp incision, a small burr hole located 2 mm to the right and 1 mm anterior to the bregma was drilled into the skull (AP:1.0 mm, ML:-2.0 mm). The syringe needle was inserted at a depth of 3.0 mm and then raised 0.4 mm to get to a depth of 2.6 mm. 2 μL of cell (2 x10^4^ cells) was injected using a 10 μL syringe (Hamilton, USA) connected to a syringe pump (KD Scientific, US; injection rate: 0.5 μL/min). After injection, the needle was left in place for an additional 5 min to avoid backflow. The burr hole was sealed with dental resin (Provinice; Shofu, Kyoto, Japan) and the skin was closed with surgical sutures. The endpoints of the cancer transplantation experiment were weight loss of 20% or more from the start of the experiment and debilitation.

#### MRI

All the MR experiments were performed at the Medical Research Support Center, Graduate School of Medicine, Kyoto University, Japan. After the induction of anesthesia with 2.5% isoflurane in air, each mouse was placed prone on the animal cradle. The anesthesia was kept with an inhalation of 2.0-3.0% isoflurane in air at flow rate of 1L/min through a face mask. The head of each mouse was fixed to the cradle using a bite-bar and surgical tape. The respiratory rate and rectal temperature of the animals were continuously monitored using a dedicated system (Model 1025, MR-compartible Small Animal Monitoring & Gating System; SA Instruments, Inc., NY, USA) and software (PC-SAM V8.04; SA Instruments). Body temperature was maintained by the flow of warm air using a heater system (MR-compatible small animal heating system; SA Instruments). MRI was performed using a 7 Tesla preclinical scanner (BioSpec 70/20 USR; Bruker BioSpin MRI GmbH, Ettlingen, Germany). A circular polarized volume coil with an inner diameter of 35 mm (T9988, Bruker BioSpin) was used for signal transmission and reception. MRI data were acquired using ParaVision 5.1 software (Bruker BioSpin). Two-dimensional multi-slice T2-weghted MR images of the whole brain were acquired using rapid acquisition with a relaxation enhancement (RARE) sequence in coronal orientation. Acquisition details were as follows: echo time (TE), 11 ms; effective TE, 33 ms; RARE factor, 8; repetition time (TR), 3000 ms; field of view (FOV), 20 mm × 20 mm; matrix size, 200 × 200; in-plane spatial resolution: 0.1 mm x 0.1 mm, number of slices, 28; slice thickness: 0.5 mm, slice gaps, 0 mm; number of averages (NA), 16; scan time, 20 min.

#### Histopathological examination

Mice were sacrificed under deep anesthesia then all organs were isolated and immediately immersed in 10% buffered neutral formalin. After fixation for 48 h, tissues were processed as formalin-fixed paraffin-embedded blocks and sliced into 3 μmμ sections, followed by hematoxylin and eosin (HE) staining, periodic acid Schiff (PAS) staining, and Elastica-Masson Goldner staining. All slides were analyzed by a skilled pathologist (T.M.). Glomerular lesions were evaluated using the histological glomerular index, and other organs were scored from 0 (normal) to 3 (necrotic, severe organ injury).

#### Western blotting

Brain tissue was collected after the mice were deeply anesthetized and perfused with PBS to remove blood. They were immediately frozen in liquid nitrogen and homogenized in Nervous Tissue Protein Extraction buffer (TCI, Tokyo, Japan). After centrifuging the lysate at 20,000 g for 10 min at 4°C, heated at 98°C for 3 min with NuPAGE Sample Reducing Agent (Thermo Fisher Scientific) and NuPAGE LDS Sample buffer (Thermo Fisher Scientific), the supernatant was used for SDS-PAGE. SDS-PAGE was performed using a 5–12% polyacrylamide gel (FUJIFILM Wako) and NuPAGE MOPS SDS running buffer (Thermo Fisher Scientific) for 1 h, 120 V. One microgram of protein was loaded in each lane. After electrophoresis, proteins were transferred to Immobilon PVDF membranes (Merck, Darmstadt, Germany) in transfer buffer (25 mM Tris, 192 mM glycine, 20% methanol) using Transblot SD cell (Bio-Rad, CA, US). After blocking the membranes with 5% skim milk in PBST (137 mM NaCl, 2.7 mM KCl, 10 mM Na_2_HPO_4_, 2 mM KH_2_PO_4_, 0.1% Tween 20) for 1 h at 20°C–25°C, they were soaked in Can Get Signal 1 (TOYOBO) with primary antibodies and incubated overnight at 4°C. After three washes with PBST, the membranes were incubated with HRP-conjugated secondary antibodies prepared in Can dGet Signal 2 (TOYOBO) for 1 h at 20°C–25°C. After three washes with PBST, the secondary antibodies were detected using ImmunoStar (FUJIFILM Wako) and LAS 3000 (FUJIFILM). The antibodies used are listed in key resources table.

### Quantification and statistical analysis

All statistical analyses were performed using GraphPad Prism 10.3.1 (GraphPad Software, San Diego, CA, USA). The statistical methods used, error bars, and sample sizes are annotated in each figure legend. Differences were considered significant when the *p*-value was less than 0.05.

## Supplemental information

Document S1. Figures S1–S12 and Tables S1–S6

Table S7. List of plasmid sequences used in this study
