# Supplementary material for: Differential substrate degradation by super-degron: EGFP in wild-type mouse cells, PD-1 requires CRBN humanization
Source: iScience. 2025 Jun 23;28(7):112992. doi: 10.1016/j.isci.2025.112992 (PMC12274739; doi:10.1016/j.isci.2025.112992)
Supplement: Document S1. Figures S1–S12 and Tables S1–S6 [file mmc1.pdf]

iScience, Volume *28*

Supplemental information

Differential substrate degradation by super-degron:

EGFP in wild-type mouse cells,

PD-1 requires CRBN humanization

Chie Naruse, Ojiro Ishibashi, Masatoshi Ohgushi, Hirohiko Imai, Tomoko Matsuzaki, Xuchi Pan, Tatsuhiko Miyazaki, Yuka Shidahara, Yu Shirakawa, Fumihiro Sugiyama, and Masahide Asano

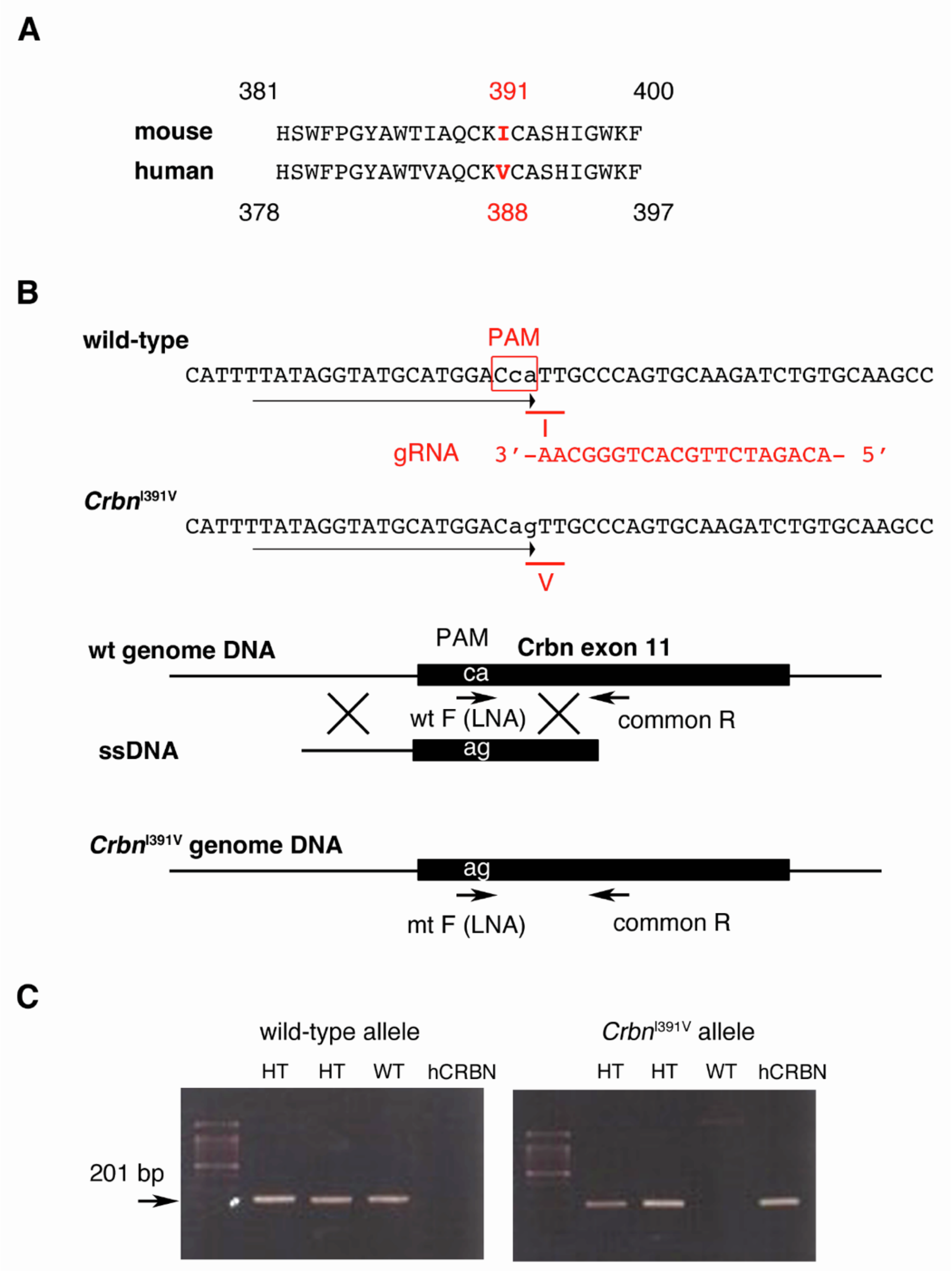

Figure S1 Generation of CRBN-humanized mice

A Amino acid sequences of mouse CRBN (upper) and human CRBN (lower) that include critical amino acids (red) for binding to thalidomide analogs.

B The DNA sequences of mouse wild-type and mutant *Crbn* (*Crbn*^{I391V}) gene for genome-editing (upper) and the schematic diagram of single-stranded (ss) DNA for homologous recombination to generate *Crbn*^{I391V} mice. PCR primers are shown (arrows). The two bases at the 3′ end of the forward primers (wt F and mt F) are locked nucleic acid (LNA) to distinguish the two-base mutations. PAM: protospacer adjacent motif, gRNA: guide RNA.

C Genotypes of wild-type and *Crbn*^{I391V} alleles determined by PCR. WT: wild-type, HT heterozygous mutant, MT homozygous mutant.

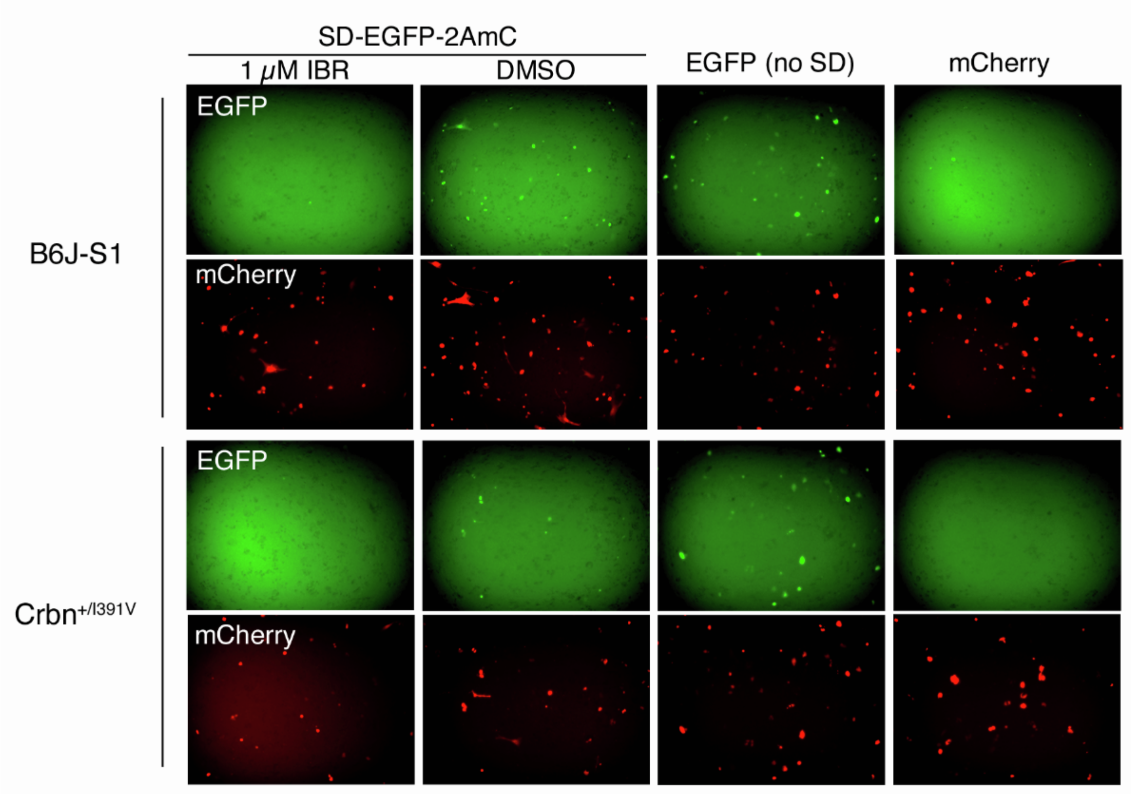

Figure S2 Super degron-tagged EGFP and mCherry images with or without IBR

EGFP and mCherry images with or without addition of 1 μM IBR in *Crbn*$^{+/+}$ B6J-S1 and *Crbn*$^{+/I391V}$ mouse embryonic stem cells 1 day after transfection with the CAGp-SD-EGFP-2A-mCherry-2A-Neo-pA (SD-EGFP-2AmC), CAGp-EGFP-2A-mCherry-2A-Neo-pA (no SD) and CAGp-mCherry-2A-Neo-pA (mCherry) vectors.

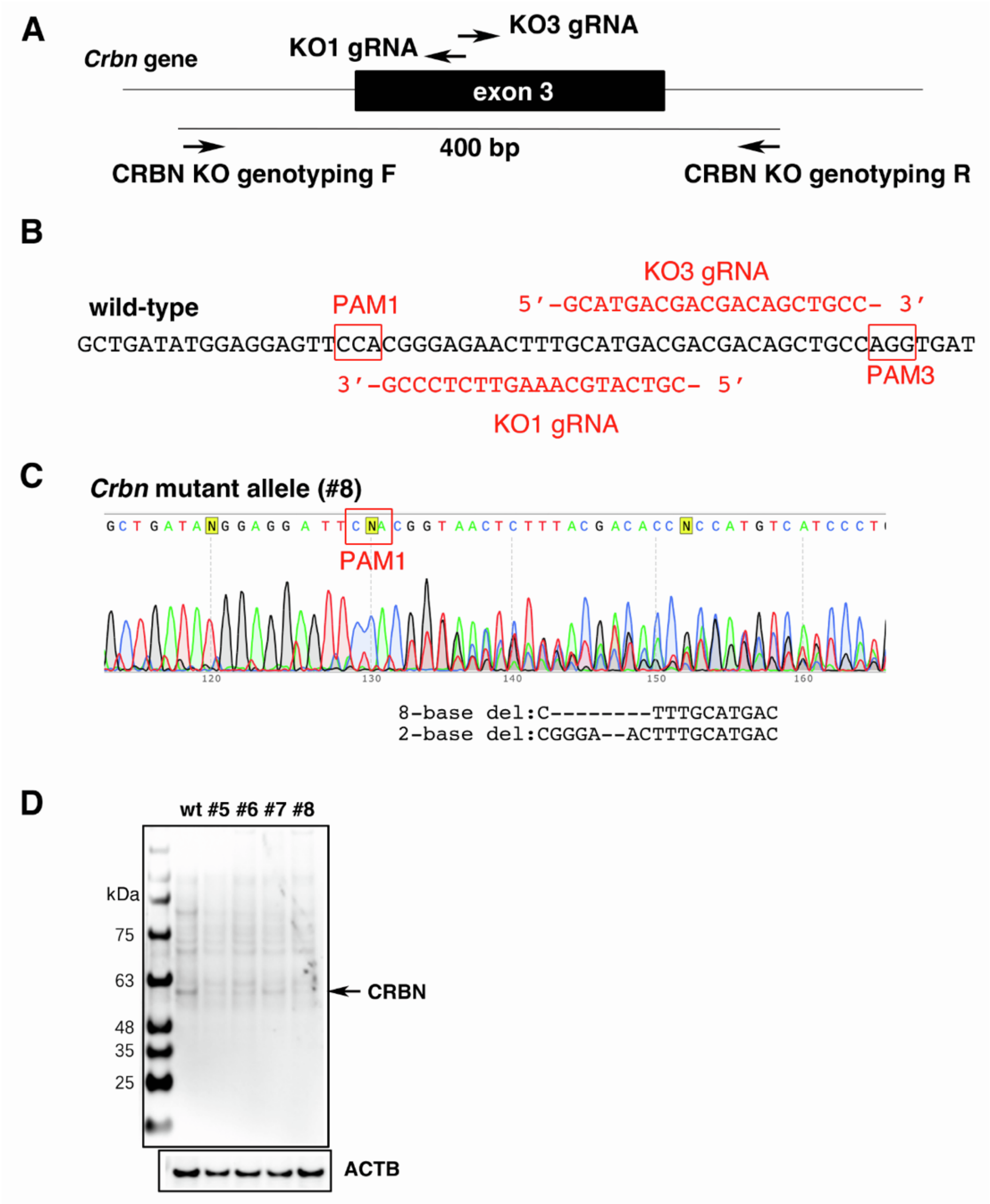

Figure S3 Generating *Crbn*$^{-/-}$ mouse embryonic stem cells by genome-editing

A The guide RNAs (KO1 gRNA and KO3 gRNA) in exon 3 of *Crbn* gene important for catalytic activity were generated for genome-editing in mouse embryonic stem cells (mESCs). PCR primers for genotyping are also shown.

B The sequences of gRNAs on wild-type *Crbn* gene. PAM: protospacer adjacent motif.

C The example of Sanger sequence result of *Crbn* mutant allele of #8 clone. PCR products synthesized from DNA derived from genome-edited mESC clone and primers indicated in **A** were used for sequencing. Wild-type sequences are also detected at low intensity due to feeder cell contamination or wild-type allele. Clone #5-#7 (not shown) and #8 had two genome-edited alleles and one wild-type allele with low signal.

D Western blotting of mESC lysate using anti-mouse CRBN antibody and anti-beta-actin (ACTB). Clone #5 and #8 showed little signal of CRBN and used for analyses in Figure 2**J**. wt: wild-type ESCs.

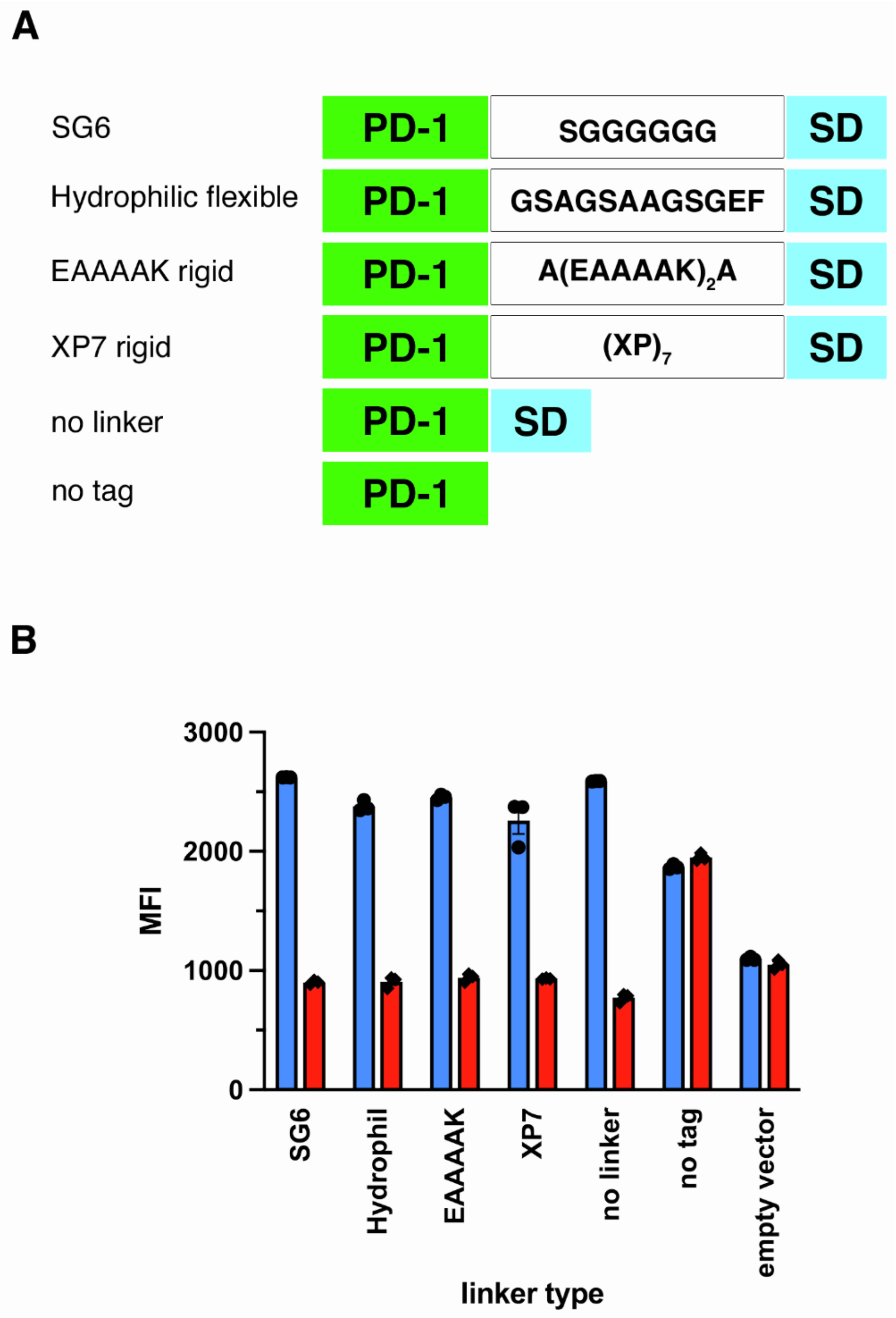

Figure S4 Evaluation of linker sequences between PD-1 and super degron tag

A Amino acid sequences of various linkers between PD-1 and super degron (SD) tag. Sequences of vectors are shown in Table S7.

B Expression amount of PD-1 with 1 μM iberdomide for 1 day in Jurkat cells. Intensities of PD-1 were measured by flow cytometry, and the geometric means of fluorescence intensities (MFI) were calculated. The dots indicate the results of the experiment for each of the three samples. Data are represented as mean ± standard deviation.

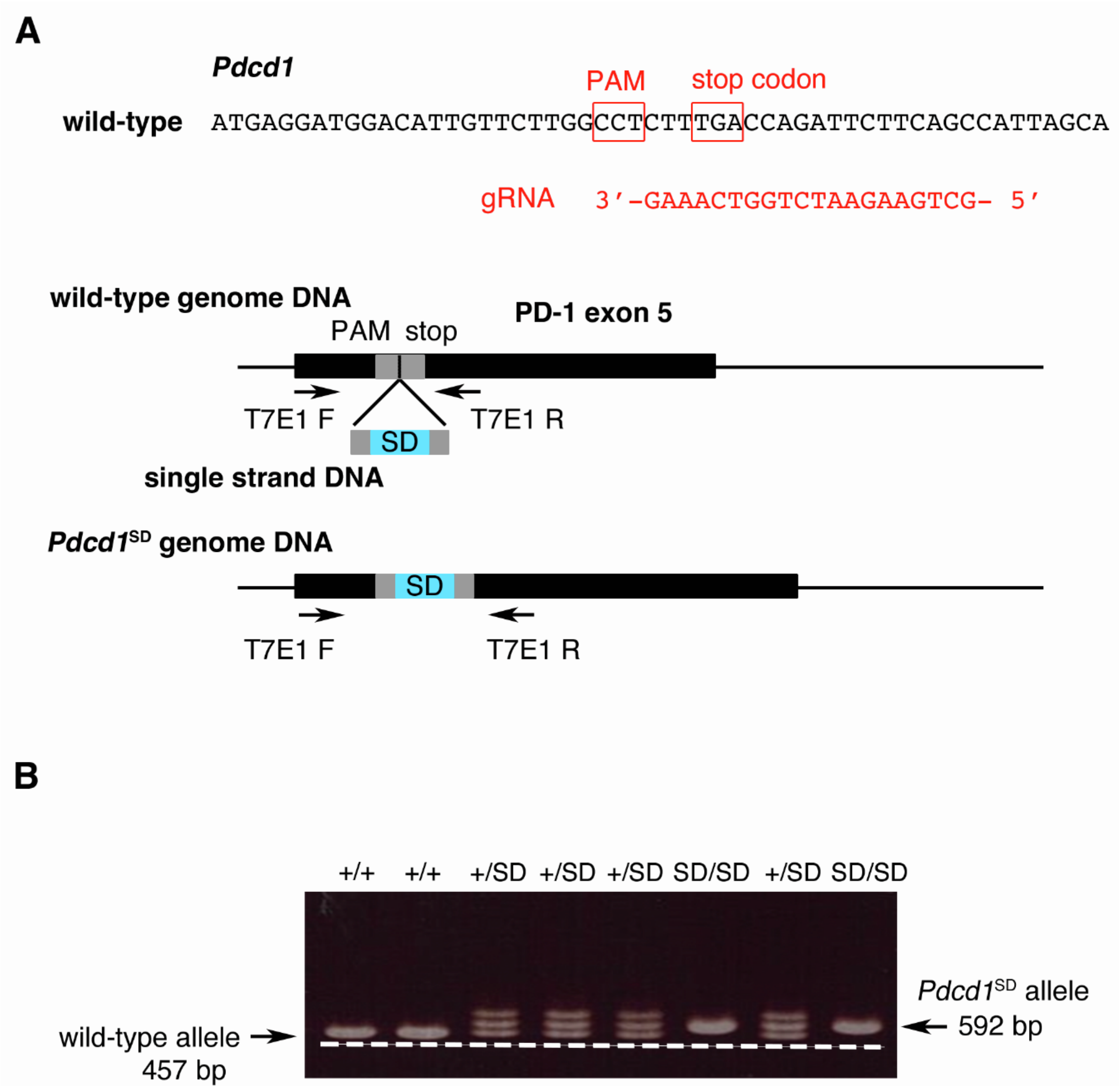

Figure S5 Generation of super degron tag-knockin mice at the PD-1 locus

A DNA sequences of mouse *Pdcd1* coding PD-1 and schematic diagram of homologous recombination for producing *Pdcd1*$^{SD/SD}$ mice. The grey rectangles in genome and single-stranded DNA oligomer are homology arms for homologous recombination. PCR primers for genotyping (T7E1 F and T7E1 R) are also shown. PAM: protospacer adjacent motif, gRNA: guide RNA.

B Genotypes of wild-type and SD knockin-Pdcd1 (*Pdcd1*^{SD}) alleles determined by PCR. Dashed lines indicate electrophoretic mobility of the wild-type allele. +/+: wild-type (WT), +/SD: heterozygous mutant, SD/SD: homozygous mutant. The DNA sequence of the PCR product was first determined by Sanger sequencing, confirming that the sequence was amplified as expected. The heterozygous mutant had three bands; the top and middle bands were found to be *Pdcd1*^{SD} alleles by Sanger sequencing and the bottom bands were wild-type.

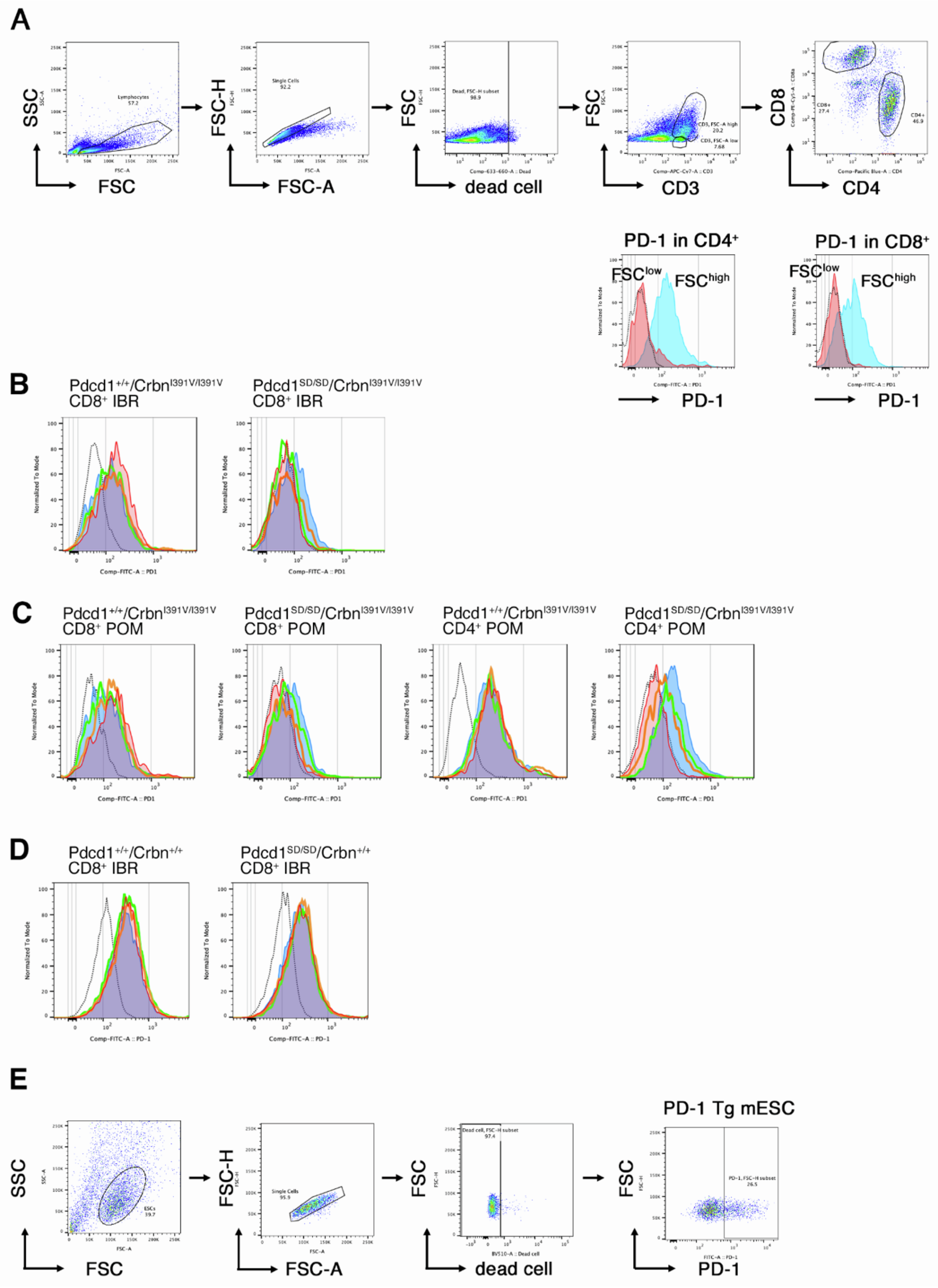

Figure S6 PD-1 expression analysis by flow cytometry

A Analyses of PD-1 expression in concanavalin-A-stimulated CD4^{+} and CD8^{+} T cells by flow cytometry.

B Typical examples of expression amount of PD-1 on CD8^{+} T cells of *Pdcd1*$^{+/+}$/*Crbn*$^{I391V/I391V}$ (left) and *Pdcd1*$^{SD/SD}$/*Crbn*$^{I391V/I391V}$ (right) with iberdomide (IBR).

C Typical examples of expression amount of PD-1 on CD8^{+} T cells of *Pdcd1*$^{+/+}$/*Crbn*$^{I391V/I391V}$ and

Pdcd1$^{SD/SD}$/*Crbn*$^{I391V/I391V}$, and that on CD4^{+} T cells of *Pdcd1*$^{+/+}$/*Crbn*$^{I391V/I391V}$ and *Pdcd1*$^{SD/SD}$/*Crbn*$^{I391V/I391V}$ with pomalidomide (POM).

D Typical examples of expression amount of PD-1 on CD8^{+} T cells of *Pdcd1*$^{+/+}$/*Crbn*$^{+/+}$ (left) and *Pdcd1*$^{SD/SD}$/*Crbn*$^{+/+}$ (right) with IBR. CD8^{+} T cells are the same as used in Figure 6E and 6F.

E Analyses of PD-1 expression on exogenous PD-1-SD-expressed mESCs by flow cytometry. The concentration of POM or IBR is 0 (blue), 10^{-3} (green), 10^{-2} (orange) and 10^{-1} μM (red). The dotted lines show isotype control without IBR. The black lines in left panels show the results of isotype control.

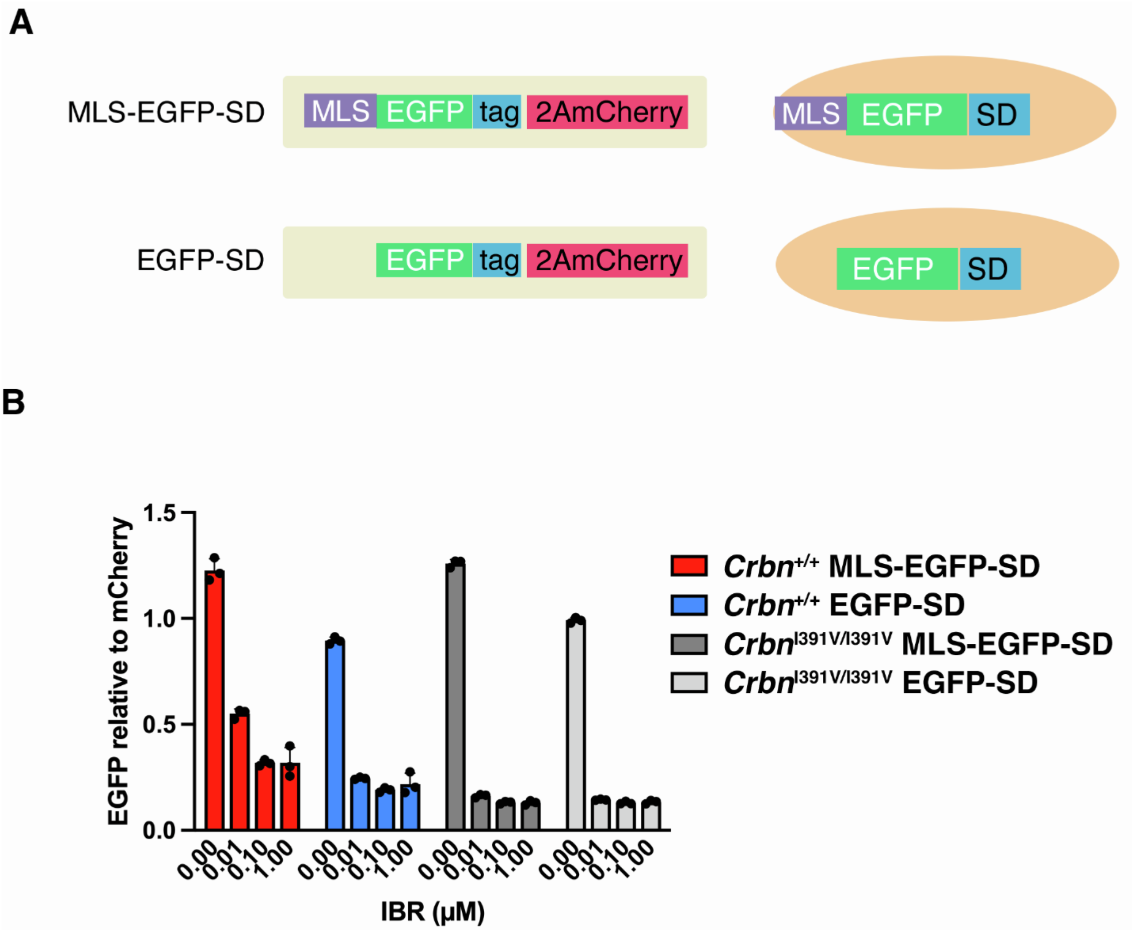

Figure S7 Effect of plasma membrane localization on EGFP-super degron tag degradation

A (left) DNA constructs for expressing EGFP-super degron tag (SD) with N-terminal 20 amino acids membrane localizing signal (MLS) from Neuromodulin derived from Addgene plasmid #57992 (mTagRFP-Membrane-1) were transfected into mouse embryonic stem cells. (right) Schema of localization of MLS-EGFP-SD and EGFP-SD in cells.

B EGFP intensity measurements and analyses are the same as in Figure 2. The dots indicate the results of the experiment for each of the three samples. Data are represented as mean ± standard deviation.

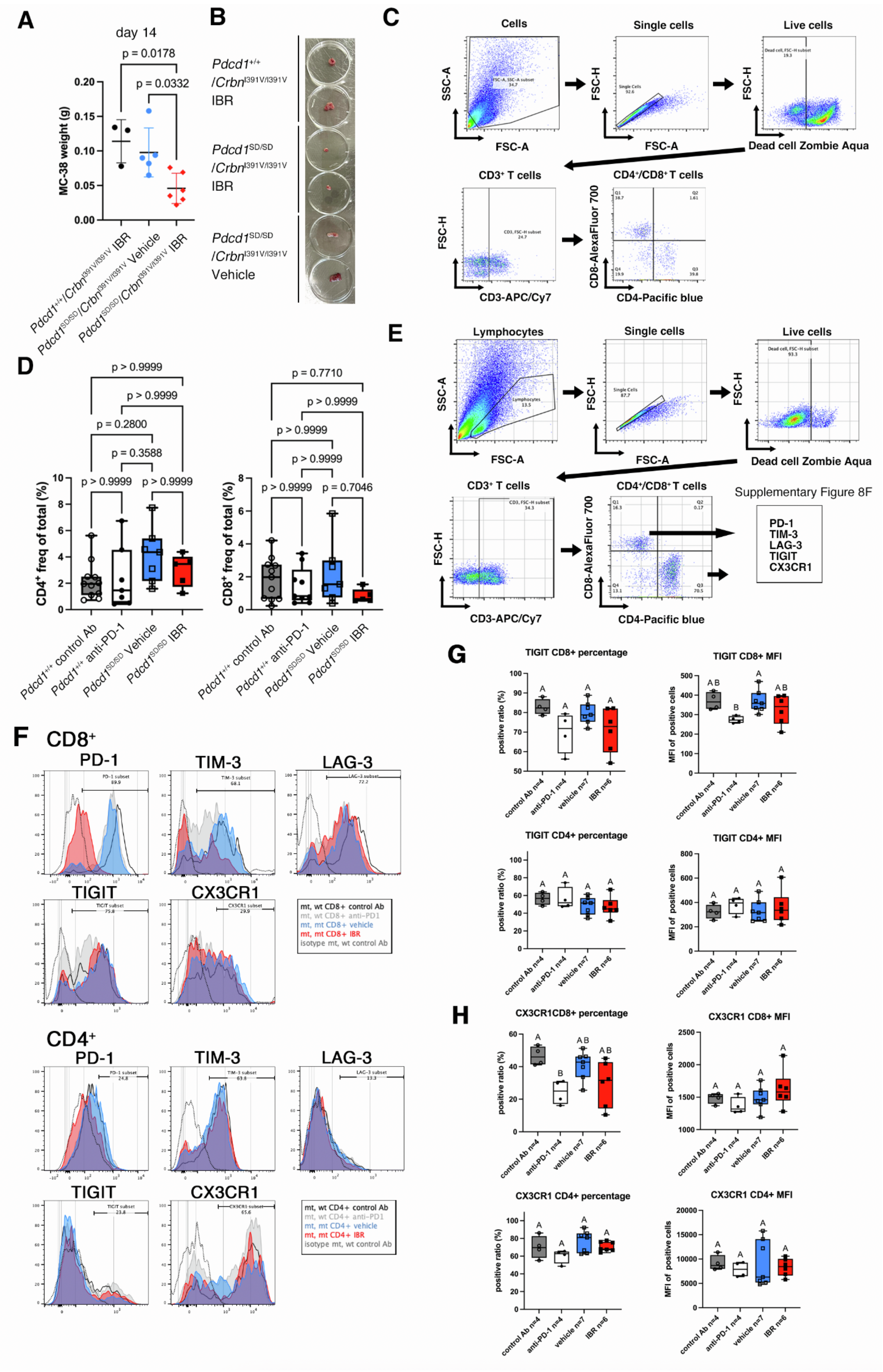

Figure S8 Growth of MC-38 and analysis of T cell markers by flow cytometry

A, B Weight (**A**) and photograph (**B**) of MC-38 clumps isolated from mice 14 days after initiation

of IBR treatment in a separate experiment from Figure 7A. *P*-values were determined by one-way ANOVA with Tukey's multiple comparisons. IBR: IBR-treated.

C, D Analyses of frequency $CD4^+$ and $CD8^+$ T cells of total cells by flow cytometry. Cells from microenvironments including MC-38 cells and whole immune cells were sorted by indicated gates. Samples are the same as Figure 7D. *Crbn*$^{I391V/I391V}$ mice were used for the experiments. Only PD-1 genotypes were shown in **D**. *Pdcd1*$^{+/+}$/*Crbn*$^{I391V/I391V}$ control Ab: n = 11, *Pdcd1*$^{+/+}$/*Crbn*$^{I391V/I391V}$ anti-PD-1 Ab n = 6, *Pdcd1*$^{SD/SD}$/*Crbn*$^{I391V/I391V}$ vehicle: n = 7, *Pdcd1*$^{SD/SD}$/*Crbn*$^{I391V/I391V}$ IBR: n = 6.

E Expression analyses of PD-1, TIM-3, LAG-3, TIGIT and CX3CR1 on T cells by flow cytometry. Cells in microenvironments were sorted by indicated gates. Dotted lines indicate isotype control.

F Typical examples of marker expressions on $CD8^+$ and $CD4^+$ T cells in microenvironments analyzed by flow cytometry.

G, H Mean fluorescence intensity of TIGIT (**G**) and CX3CR1 (**H**) on $CD4^+$ and $CD8^+$ T cells isolated from tumor microenvironments implanted with MC-38 9 days after initiation of IBR or anti-PD-1 treatment. *P*-values were determined by Kruskal-Wallis test. Different letters indicate statistically significant differences between groups (p < 0.05). Box plots: hinges, 25th and 75th percentiles; middle line, median; whiskers, minimum to maximum value.

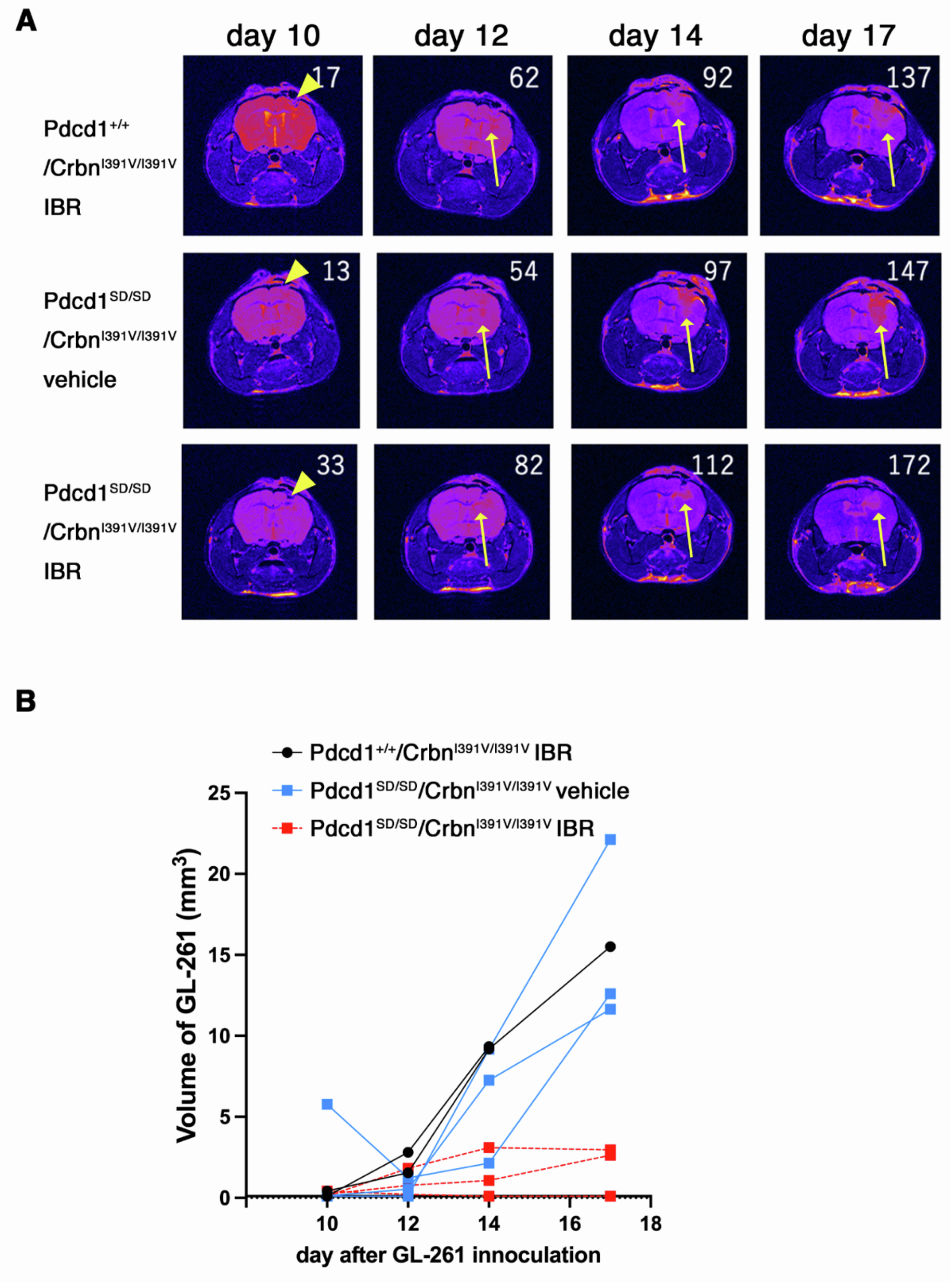

Figure S9 Growth of GL-261 in *Pdcd1*$^{SD/SD}$/*Crbn*$^{I391V/I391V}$ brains with or without iberdomide

A Images for analyses of proliferation of GL-261 cells in *Pdcd1*$^{+/+}$/*Crbn*$^{I391V/I391V}$ brains with iberdomide (IBR) (top), *Pdcd1*$^{SD/SD}$/*Crbn*$^{I391V/I391V}$ without IBR (middle) and *Pdcd1*$^{SD/SD}$/*Crbn*$^{I391V/I391V}$ with IBR obtained by magnetic resonance imaging (MRI). The number in each image indicates the serial number of images for analyses. Arrowheads: injured tissue by needle for injection. Arrows: expanded GL-261 cells in brains.

B Growth of GL-261 cells in each mouse brain (*Pdcd1*$^{+/+}$/*Crbn*$^{I391V/I391V}$ with IBR: n = 2, *Pdcd1*$^{SD/SD}$/*Crbn*$^{I391V/I391V}$ with vehicle: n = 3, *Pdcd1*$^{SD/SD}$/*Crbn*$^{I391V/I391V}$ with IBR: n = 3). The number of *Pdcd1*$^{+/+}$/*Crbn*$^{I391V/I391V}$ mice is one at day 17 because one mouse died on day 16.

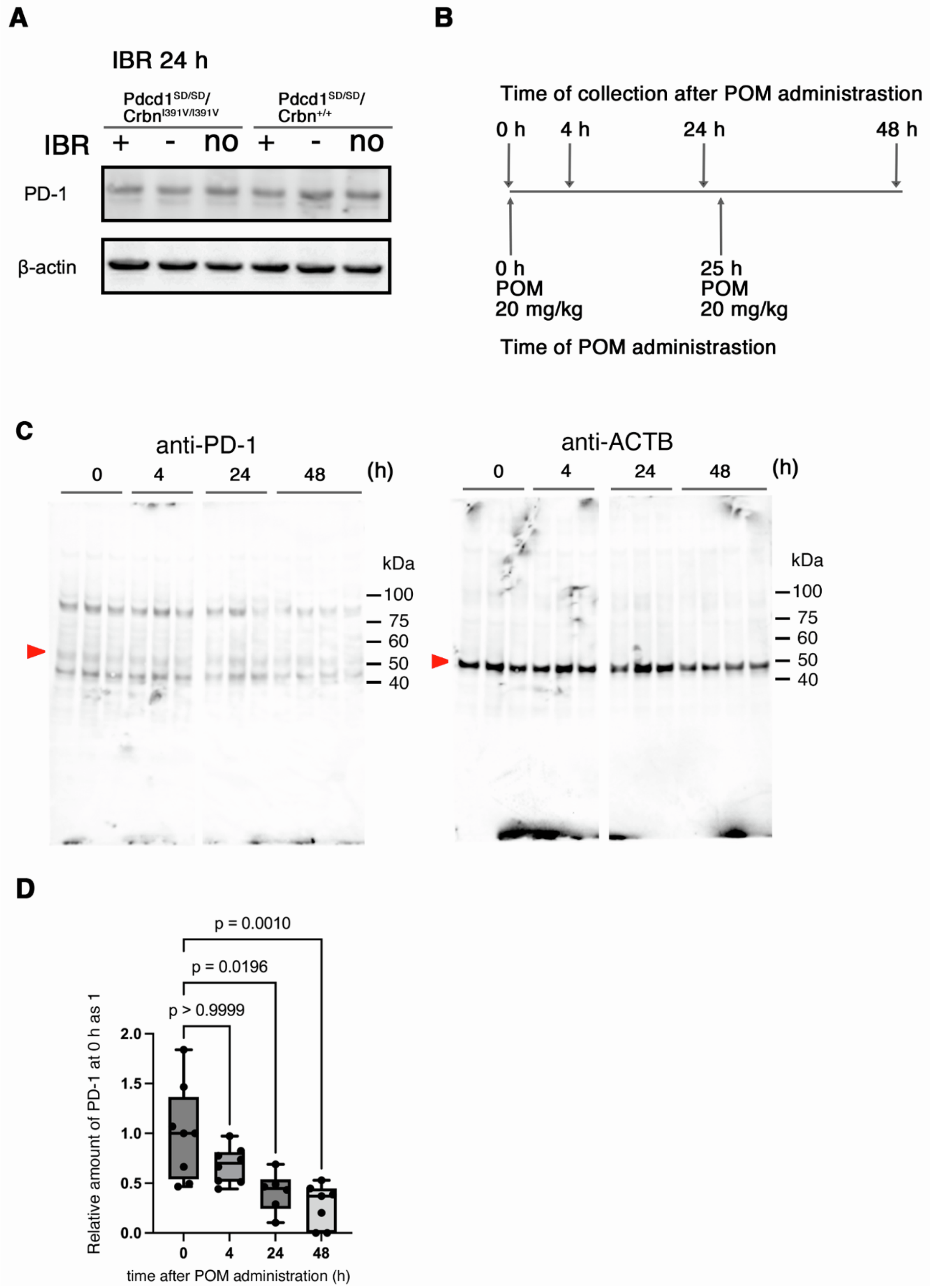

Figure S10 PD-1 expression levels in *Pdcd1*$^{SD/SD}$/*Crbn*$^{I391V/I391V}$ brains

A Western blotting analysis of PD-1 in *Pdcd1*$^{SD/SD}$/*Crbn*$^{I391V/I391V}$ and *Pdcd1*$^{SD/SD}$/*Crbn*$^{+/+}$ brains. Iberdomide (IBR) (10 mg/kg) was administered, and brains were collected at 24 h after treatment. +: IBR, -: vehicle, no: no treatment.

B The time course of pomalidomide (POM) administration and brain collection. POM (20 mg/kg) was administered at 0 h and 25 h. Brains were collected without POM administration (0 h), after

POM administration once (4 h and 24 h) and twice (48 h).

C Western blotting analysis of PD-1 in *Pdcd1*$^{SD/SD}$/*Crbn*$^{I391V/I391V}$ brains with (4, 24, 48 h) or without (0 h) POM. Arrowheads show the specific bands for PD-1 and beta-ACTIN (ACTB). The calculated molecular weight of PD-1 is 32 kDa, however, PD-1 appears to have a molecular mass of 45-55 kDa on SDS-PAGE because of glycosylation.[1,2] The calculated molecular weight of ACTB is 42 kDa.

D PD-1 expression levels in *Pdcd1*$^{SD/SD}$/*Crbn*$^{I391V/I391V}$ brains. The relative amount of PD-1 was standardized by ACTB and the relative amount of PD-1 at time 0 was set to 1. The results of three experiments are combined. 0 and 4 h: n = 8, 24 h: n = 6, 48 h: n = 7.

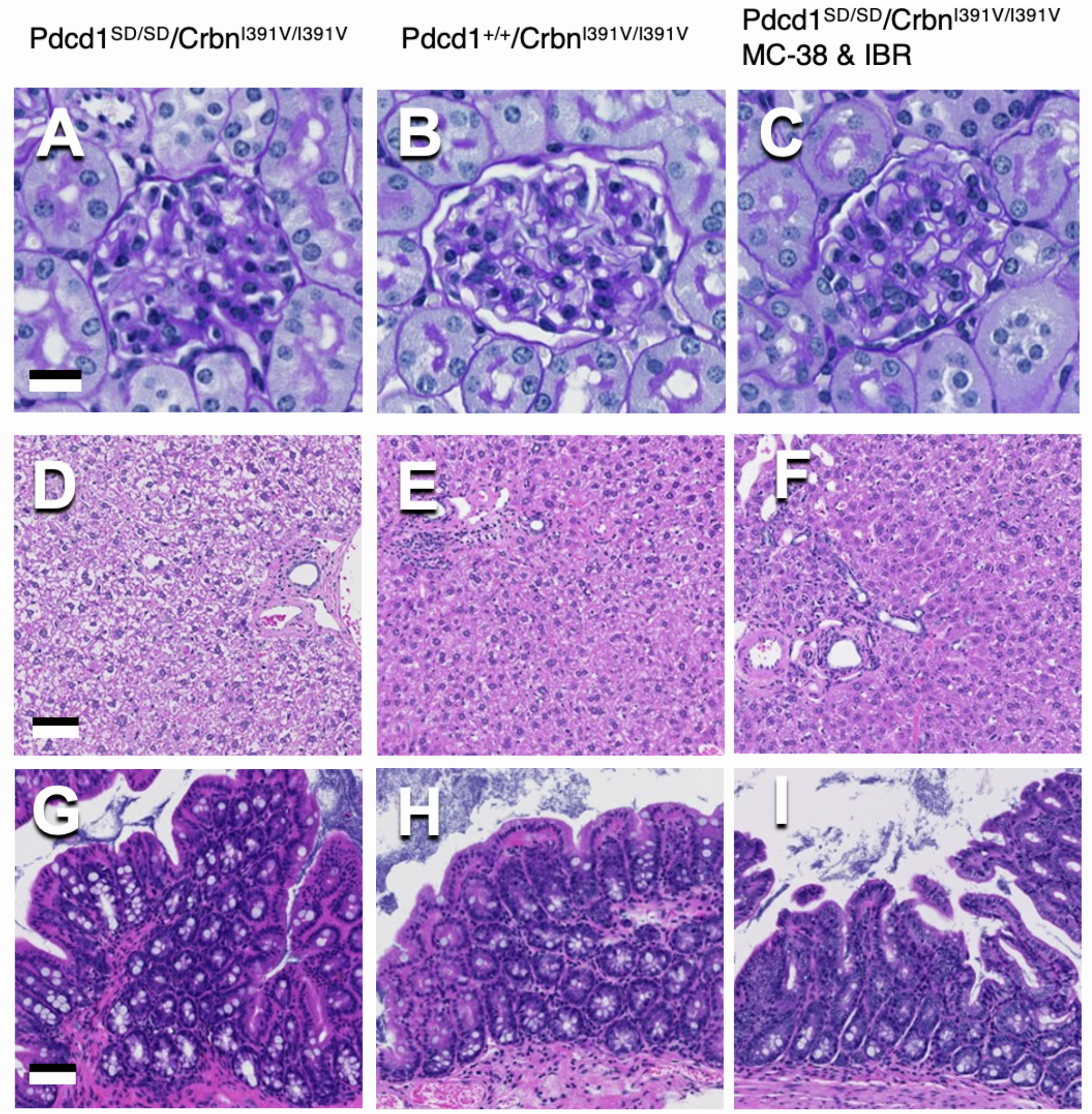

Figure S11 Histological analysis of super degron tag-knockin mice for autoimmune disease

Representative histological manifestations of kidney (**A-C**), liver (**D-F**) and large intestine (**G-I**) of *Pdcd1*$^{SD/SD}$/*Crbn*$^{I391V/I391V}$ mice (**A, D** and **G**), *Pdcd1*$^{+/+}$/*Crbn*$^{I391V/I391V}$ mice (**B, E** and **H**) and *Pdcd1*$^{SD/SD}$/*Crbn*$^{I391V/I391V}$ mice with MC-38 inoculation and IBR treatment (**C, F** and **I**). Nevertheless, a few glomeruli in *Pdcd1*$^{+/+}$/*Crbn*$^{I391V/I391V}$ mice showed slight mesangial matrix hyperplasia, glomeruli in all group mice showed histological manifestations within normal limits. Liver and large intestine manifested normal histological findings in each group (**A-C**: PAS stain, d-i: HE stain, Bar = 25 μm in **A-C**, 100 μm in **D-I**).

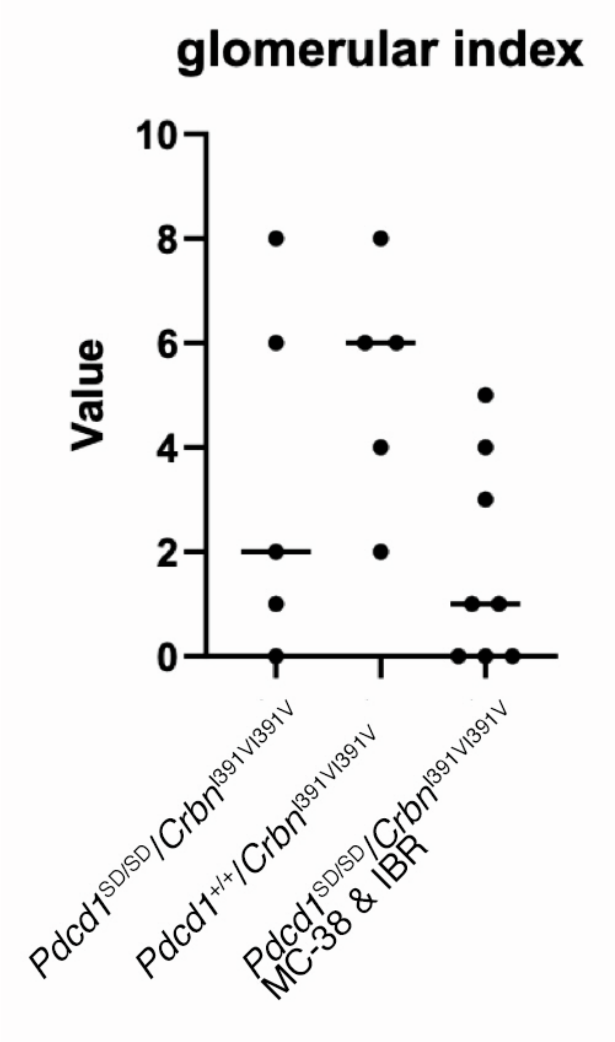

Figure S12 Glomerular index of super degron tag-knockin mice

Glomerular index of each group of mice. *Pdcd1*$^{+/+}$/*Crbn*$^{I391V/I391V}$ mice showed slight higher index nevertheless there were no statistically significant differences among groups.

Related to Figure 1C									
log(inhibitor) vs. response (three parameters)									
Best-fit values	IKZF min FITC	IKZF full FITC	SALL4 FITC	SD (ZFP91/IZ) FITC		IKZF min mC	IKZF full mC	SALL4 mC	SD (ZFP91/IZ) mC
Bottom	0.5295	0.3218	0.9333	0.1083		0.8201	0.6165	Unstable	0.2421
Top	1	1	1	1		1	1	3.526E+73	1
LogDC50 (μM)	0.1515	-0.3826	Unstable	-0.8287		0.04896	-0.49	Unstable	-0.631
DC50 (μM)	1.417	0.4143	Unstable	0.1483		1.119	0.3236	Unstable	0.2339
Span	0.4706	0.6782	0.0668	0.8917		0.18	0.3835	Unstable	0.7579
95% CI (profile likelihood)									
Bottom	-0.02930 to 0.6877	0.05036 to 0.4445	??? to 1.336	0.07044 to 0.1456		???	??? to 0.7801	(Very wide)	0.2078 to 0.2758
Top	0.8295 to 1.171	0.8387 to 1.161	0.5640 to ???	0.9685 to 1.032		0.7205 to 1.349	0.7402 to 1.260	???	0.9726 to 1.027
LogDC50 (μM)	??? to 1.348	??? to 0.6648	(Very wide)	-0.9727 to -0.7105		???	???	(Very wide)	-0.7443 to -0.5300
DC50 (μM)	??? to 22.30	??? to 4.622	(Very wide)	0.1065 to 0.1947		???	???	(Very wide)	0.1802 to 0.2951
Goodness of Fit									
Degrees of Freedom	1	1	1	6		1	1	1	6
R squared	0.9986	0.9995	0.4731	0.9976		0.9627	0.9959	-1.23E+150	0.9973
Sum of Squares	0.0001805	0.0001612	0.003713	0.002985		0.0007528	0.0004178	4.973E+147	0.002259
Sy.x	0.01343	0.0127	0.06093	0.0223		0.02744	0.02044	7.052E+73	0.0194
Number of points									
# of X values	15	15	15	9		15	15	15	9
# Y values analyzed	4	4	4	9		4	4	4	9

Related to Figure 1D									
log(inhibitor) vs. response (three parameters)									
Best-fit values	SD IBR FITC	SD POM FITC				SD POM mC	SD IBR mC		
Bottom	0.1258	0.1083				0.2421	0.2415		
Top	1.001	1				1	1		
LogDC50 (μM)	-6.782	-0.8287				-0.631	-2.495		
DC50 (μM)	1.651E-07	0.1483				0.2339	0.003196		
Span	0.8747	0.8917				0.7579	0.7585		
95% CI (profile likelihood)									
Bottom	0.1044 to 0.1400	0.07044 to 0.1456				0.2078 to 0.2758	0.2220 to 0.2563		
Top	???	0.9685 to 1.032				0.9726 to 1.027	0.9826 to 1.017		
LogDC50 (μM)	???	-0.9727 to -0.7105				-0.7443 to -0.5300	??? to -1.662		
DC50 (μM)	???	0.1065 to 0.1947				0.1802 to 0.2951	??? to 0.02176		
Goodness of Fit									
Degrees of Freedom	6	6				6	6		
R squared	0.9992	0.9976				0.9973	0.9992		
Sum of Squares	0.001207	0.002985				0.002259	0.0009067		
Sy.x	0.01418	0.0223				0.0194	0.01229		
Number of points									
# of X values	9	9				9	9		
# Y values analyzed	9	9				9	9		

*Fluorescence intensities were standardized with the sample to which no reagent was added as 1.
*The concentrations of 0 μM were analyzed as -10 μM.

Table S1 The best-fit values of response curves related to Figure 1

Related to Figure 2C SD-EGFP

log(inhibitor) vs. response (three parameters)				
Best-fit values	B6J-S1	mCRBN	h/mCRBN	hCRBN
Bottom	0.09529	0.113	0.07126	0.04953
Top	0.3893	0.4455	0.3897	0.3789
LogDC50 (μM)	-2.739	-2.217	-4.106	-4.196
DC50 (μM)	0.001824	0.006063	0.00007839	0.00006375
Span	0.294	0.3325	0.3185	0.3294
95% CI (profile likelihood)				
Bottom	0.07925 to 0.1109	0.08261 to 0.1425	0.04590 to 0.09630	0.03172 to 0.06718
Top	0.3772 to 0.4015	0.4270 to 0.4642	0.3593 to 0.4209	0.3568 to 0.4015
LogDC50 (μM)	-2.900 to -2.570	-2.456 to -1.994	-4.388 to -3.826	-4.389 to -4.003
DC50 (μM)	0.001259 to 0.002692	0.003498 to 0.01013	4.095e-005 to 0.00014	4.079e-005 to 9.930e-005
Goodness of Fit				
Degrees of Freedom	21	21	21	21
R squared	0.9805	0.9551	0.9338	0.9673
Sum of Squares	0.007697	0.02122	0.03278	0.01658
Sy.x	0.01915	0.03179	0.03951	0.0281
Number of points				
# of X values	24	24	24	24
# Y values analyzed	24	24	24	24
bottom vs top	0.24	0.25	0.18	0.13

Related to Figure 2D EGFP-SD

log(inhibitor) vs. response (three parameters)				
	B6J-S1	mCRBN	h/mCRBN	hCRBN
Bottom	0.03557	0.04584	0.01502	0.007275
Top	0.3429	0.3045	0.3487	0.2738
LogDC50 (μM)	-3.089	-2.664	-4.151	-4.185
DC50 (μM)	0.0008154	0.002167	0.00007071	0.00006538
Span	0.3073	0.2586	0.3337	0.2665
95% CI (profile likelihood)				
Bottom	0.02856 to 0.04254	0.04009 to 0.05154	0.009373 to 0.02066	0.001091 to 0.01344
Top	0.3368 to 0.3489	0.3002 to 0.3088	0.3417 to 0.3557	0.2661 to 0.2816
LogDC50 (μM)	-3.159 to -3.019	-2.730 to -2.598	-4.212 to -4.089	-4.272 to -4.098
DC50 (μM)	0.0006939 to 0.00095	0.001863 to 0.002526	6.133e-005 to 8.143e-	5.340e-005 to 7.976e-005
Goodness of Fit				
Degrees of Freedom	21	21	21	21
R squared	0.996	0.9967	0.9968	0.9939
Sum of Squares	0.00177	0.0009763	0.001644	0.001968
Sy.x	0.009182	0.006818	0.008849	0.009682
Number of points				
# of X values	24	24	24	24
# Y values analyzed	24	24	24	24
	0.10	0.15	0.04	0.03

*The concentrations of samples to which no POM or IBR was added were analyzed as -10 μM.

Table S2 The best-fit values of response curves related to Figure 2

Related to Figure 3A

log(inhibitor) vs. response (three parameters)			
Best-fit values	SD-EGFP mouse ESCs	SD-EGFP rat ESCs	SD-EGFP human ESCs
Bottom	0.0299	0.01454	0.03396
Top	0.205	0.2567	0.6664
LogDC50	-3.182	-3.567	-5.898
DC50	0.0006569	0.0002708	0.000001266
Span	0.175	0.2421	0.6324
95% CI (profile likelihood)			
Bottom	0.02675 to 0.03305	0.005482 to 0.02344	0.02662 to 0.04130
Top	0.2016 to 0.2083	0.2453 to 0.2683	0.6504 to 0.6824
LogDC50	-3.241 to -3.124	-3.717 to -3.412	-6.034 to -5.788
DC50	0.0005737 to 0.0007508	0.0001918 to 0.0003871	9.242e-007 to 1.629e-006
Goodness of Fit			
Degrees of Freedom	18	18	18
R squared	0.9974	0.9881	0.9969
Sum of Squares	0.0003102	0.002716	0.003116
Sy.x	0.004151	0.01228	0.01316
Number of points			
# of X values	21	21	21
# Y values analyzed	21	21	21
bottom vs top	0.146	0.057	0.051
Bottom (mouse vs human)		0.880	
Top (mouse vs human)		0.307	
LogDC50 (mouse vs human)		519.996	

log(inhibitor) vs. response (three parameters)			
	EGFP-SD mouse ESCs	EGFP-SD rat ESCs	EGFP-SD human ESCs
Bottom	0.02573	0.0497	0.007522
Top	0.3021	0.6003	1.592
LogDC50	-3.657	-3.855	-5.556
DC50	0.0002205	0.0001396	0.000002778
Span	0.2764	0.5506	1.585
95% CI (profile likelihood)			
Bottom	0.01974 to 0.03168	0.03843 to 0.06091	-0.003457 to 0.01850
Top	0.2942 to 0.3101	0.5842 to 0.6165	1.568 to 1.616
LogDC50	-3.739 to -3.573	-3.932 to -3.775	-5.599 to -5.515
DC50	0.0001825 to 0.0002676	0.0001168 to 0.0001677	2.515e-006 to 3.052e-006
Goodness of Fit			
Degrees of Freedom	18	18	18
R squared	0.9955	0.9955	0.9989
Sum of Squares	0.001303	0.004977	0.006884
Sy.x	0.00851	0.01663	0.01956
Number of points			
# of X values	21	21	21
# Y values analyzed	21	21	21
bottom vs top	0.085	0.083	0.005
Bottom (mouse vs human)		0.292	
Top (mouse vs human)		5.270	
LogDC50 (mouse vs human)		79.250	

Related to Figure 3B

log(inhibitor) vs. response (three parameters)		
Best-fit values	SD-EGFP NIH3T3	SD-EGFP HEK293
Bottom	0.04935	0.02006
Top	0.266	0.3107
LogDC50	-3.712	-5.517
DC50	0.0001942	0.000003042
Span	0.2167	0.2906
95% CI (profile likelihood)		
Bottom	0.04744 to 0.05126	0.01758 to 0.02253
Top	0.2634 to 0.2686	0.3053 to 0.3160
LogDC50	-3.744 to -3.679	-5.568 to -5.469
DC50	0.0001801 to 0.0002096	2.706e-006 to 3.396e-006
Goodness of Fit		
Degrees of Freedom	18	18
R squared	0.9992	0.9983
Sum of Squares	0.0001386	0.000349
Sy.x	0.002775	0.004403
Number of points		
# of X values	21	21
# Y values analyzed	21	21
bottom vs top	0.186	0.065
Bottom (mouse vs human)		2.460
Top (mouse vs human)		0.856
LogDC50 (mouse vs human)		0.016

log(inhibitor) vs. response (three parameters)		
	EGFP-SD NIH3T3	EGFP-SD HEK293
Bottom	0.03706	0.01028
Top	0.7502	1.151
LogDC50	-4.103	-5.264
DC50	0.00007897	0.000005448
Span	0.7132	1.14
95% CI (profile likelihood)		
Bottom	0.02956 to 0.04455	0.006242 to 0.01431
Top	0.7386 to 0.7619	1.142 to 1.159
LogDC50	-4.141 to -4.064	-5.280 to -5.248
DC50	7.221e-005 to 8.636e-005	5.251e-006 to 5.650e-006
Goodness of Fit		
Degrees of Freedom	18	18
R squared	0.9986	0.9997
Sum of Squares	0.002418	0.0009122
Sy.x	0.01159	0.007119
Number of points		
# of X values	21	21
# Y values analyzed	21	21
bottom vs top	0.049	0.009
Bottom (mouse vs human)		3.605
Top (mouse vs human)		0.652
LogDC50 (mouse vs human)		0.069

Related to Figure 3C

log(inhibitor) vs. response (three parameters)		
Best-fit values	SD-EGFP EL4	SD-EGFP Jurkat
Bottom	0.07839	0.0763
Top	0.5126	0.5983
LogIC50	-3.718	-5.117
IC50	0.0001916	0.000007635
Span	0.4343	0.522
95% CI (profile likelihood)		
Bottom	0.05574 to 0.1005	0.06763 to 0.08494
Top	0.4884 to 0.5372	0.5830 to 0.6137
LogIC50	-3.916 to -3.504	-5.185 to -5.049
IC50	0.0001212 to 0.0003135	6.531e-006 to 8.927e-006
Goodness of Fit		
Degrees of Freedom	21	21
R squared	0.9762	0.9952
Sum of Squares	0.02153	0.004961
Sy.x	0.03202	0.01537
Number of points		
# of X values	24	24
# Y values analyzed	24	24
bottom vs top	0.153	0.128
Bottom (mouse vs human)		1.027
Top (mouse vs human)		0.857
LogDC50 (mouse vs human)		0.040

log(inhibitor) vs. response (three parameters)		
	EGFP-SD EL4	EGFP-SD Jurkat
Bottom	0.04356	0.02414
Top	0.7643	0.9917
LogDC50	-4.122	-4.929
DC50	0.00007551	0.00001178
Span	0.7207	0.9675
95% CI (profile likelihood)		
Bottom	0.03380 to 0.05329	0.01536 to 0.03292
Top	0.7489 to 0.7797	0.9734 to 1.010
LogDC50	-4.174 to -4.070	-4.967 to -4.890
DC50	6.694e-005 to 8.513e-005	1.080e-005 to 1.287e-005
Goodness of Fit		
Degrees of Freedom	18	18
R squared	0.9977	0.9984
Sum of Squares	0.004052	0.004078
Sy.x	0.015	0.01505
Number of points		
# of X values	21	21
# Y values analyzed	21	21
bottom vs top	0.057	0.024
Bottom (mouse vs human)		1.804
Top (mouse vs human)		0.771
LogDC50 (mouse vs human)		0.156

*The concentrations of samples to which no POM or IBR was added were analyzed as -10 μM.

Table S3 The best-fit values of response curves related to Figure 3

Related to Figure 4A mESCs degradation

One phase decay				
Best-fit values	0 μM	0.01 μM	0.1 μM	1 μM
Y0	1	0.9945	0.9964	0.996
Plateau	0.9999	0.1599	0.1092	0.09822
K	0.592	1.11	1.249	1.239
Half Life (h)	1.171	0.6247	0.5549	0.5593
Tau	1.689	0.9013	0.8006	0.8069
Span	0.0001088	0.8346	0.8872	0.8978
95% CI (profile likelihood)				
Y0	0.9756 to 1.024	0.9542 to 1.035	0.9600 to 1.033	0.9523 to 1.040
Plateau	0.9890 to 1.011	0.1365 to 0.1828	0.08876 to 0.1294	0.07357 to 0.1224
K	???	0.9485 to 1.305	1.091 to 1.439	1.056 to 1.467
Half Life	???	0.5310 to 0.7308	0.4819 to 0.6352	0.4725 to 0.6565
Tau	???	0.7660 to 1.054	0.6952 to 0.9164	0.6816 to 0.9471
Goodness of Fit				
Degrees of Freedom	15	15	15	15
R squared	0.000004725	0.9904	0.993	0.9903
Sum of Squares	0.005921	0.01592	0.01304	0.01871
Sy.x	0.01987	0.03258	0.02948	0.03532
Constraints				
K	K > 0	K > 0	K > 0	K > 0
Number of points				
# of X values	18	18	18	18
# Y values analyzed	18	18	18	18

Related to Figure 4B NIH/3T3 degradation

One phase decay				
Best-fit values	0 μM	0.01 μM	0.1 μM	1 μM
Y0	1	0.9991	0.9995	0.9996
Plateau	1	0.1206	0.09879	0.0887
K	Unstable	1.917	2.205	2.261
Half Life (h)	Unstable	0.3616	0.3144	0.3065
Tau	Unstable	0.5217	0.4536	0.4422
Span	0	0.8785	0.9008	0.9109
95% CI (profile likelihood)				
Y0	0.9704 to ???	0.9625 to 1.036	0.9689 to 1.030	0.9710 to 1.028
Plateau	??? to 1.013	0.09998 to 0.1408	0.08206 to 0.1153	0.07319 to 0.1040
K	(Very wide)	1.621 to 2.343	1.888 to 2.673	1.953 to 2.711
Half Life	(Very wide)	0.2959 to 0.4277	0.2593 to 0.3672	0.2556 to 0.3550
Tau	(Very wide)	0.4269 to 0.6170	0.3741 to 0.5297	0.3688 to 0.5121
Goodness of Fit				
Degrees of Freedom	15	15	15	15
R squared	0	0.9928	0.9953	0.996
Sum of Squares	0.008662	0.0133	0.009301	0.008109
Sy.x	0.02403	0.02977	0.0249	0.02325
Constraints				
K	K > 0	K > 0	K > 0	K > 0
Number of points				
# of X values	18	18	18	18
# Y values analyzed	18	18	18	18

Related to Figure 4C EL4 degradation

One phase decay				
Best-fit values	0 μM	0.01 μM	0.1 μM	1 μM
Y0	1	1	1	1
Plateau	0.9997	0.106	0.0948	0.09035
K	0.1795	1.978	2.093	2.1
Half Life (h)	3.861	0.3505	0.3312	0.3301
Tau	5.57	0.5057	0.4779	0.4762
Span	0.0006538	0.894	0.9054	0.9098
95% CI (profile likelihood)				
Y0	??? to 1.011	0.9677 to 1.032	0.9676 to 1.033	0.9766 to 1.024
Plateau	??? to 1.005	0.08815 to 0.1235	0.07709 to 0.1122	0.07754 to 0.1030
K	???	1.711 to 2.344	1.803 to 2.506	1.881 to 2.382
Half Life	???	0.2957 to 0.4050	0.2766 to 0.3845	0.2910 to 0.3684
Tau	???	0.4266 to 0.5844	0.3991 to 0.5548	0.4198 to 0.5316
Goodness of Fit				
Degrees of Freedom	15	15	15	15
R squared	0.0007678	0.9946	0.9947	0.9972
Sum of Squares	0.001058	0.01029	0.0105	0.005526
Sy.x	0.008399	0.02619	0.02645	0.01919
Constraints				
K	K > 0	K > 0	K > 0	K > 0
Number of points				
# of X values	18	18	18	18
# Y values analyzed	18	18	18	18

Related to Figure 4D mESCs recovery

raw data	0			average
0:00:00	1.023	0.987	0.99	1.000
3:00:00	1.008	1.004	0.988	1.000
5:00:00	0.994	0.994	1.012	1.000
24:00:00	1.006	1.001	0.993	1.000
	0.01			
0:00:00	0.141	0.148	0.147	0.145
3:00:00	0.713	0.682	0.667	0.687
5:00:00	0.656	0.69	0.715	0.687
24:00:00	0.67	0.657	0.666	0.664
	0.1			
0:00:00	0.092	0.089	0.09	0.090
3:00:00	0.466	0.439	0.442	0.449
5:00:00	0.321	0.379	0.389	0.363
24:00:00	0.35	0.334	0.351	0.345
	1			
0:00:00	0.081	0.09	0.061	0.077
3:00:00	0.213	0.214	0.222	0.216
5:00:00	0.152	0.165	0.161	0.159
24:00:00	0.155	0.149	0.153	0.152

Related to Figure 4E NIH/3T3 recovery

raw data	0			average
0:00:00	0.97	1	1.03	1.000
1:00:00	0.98	1.007	1.013	1.000
2:00:00	0.989	1.011	1	1.000
3:00:00	1.002	1	0.998	1.000
6:00:00	1.017	0.994	0.99	1.000
24:00:00	1.045	0.975	0.98	1.000
	0.01			
0:00:00	0.137	0.13	0.132	0.133
1:00:00	0.306	0.302		0.304
2:00:00	0.488	0.463	0.468	0.473
3:00:00	0.573	0.604	0.614	0.597
6:00:00	0.554	0.543	0.558	0.552
24:00:00	0.723	0.763	0.713	0.733
	0.1			
0:00:00	0.102	0.1	0.103	0.102
1:00:00	0.249	0.245	0.24	0.245
2:00:00	0.382	0.375	0.343	0.367
3:00:00	0.465	0.434	0.428	0.442
6:00:00	0.315	0.338	0.306	0.320
24:00:00	0.347	0.341	0.328	0.339
	1			
0:00:00	0.092	0.096	0.095	0.094
1:00:00	0.22	0.212	0.216	0.216
2:00:00	0.236	0.236	0.199	0.224
3:00:00	0.266	0.255	0.255	0.259
6:00:00	0.145	0.147	0.139	0.144
24:00:00	0.133	0.149	0.146	0.143

Related to Figure 4F EL4 recovery

raw data	0			average
0:00:00	0.976	1.006	1.017	1.000
1:00:00	1.006	1.002	0.992	1.000
2:00:00	0.998	1.005	0.997	1.000
3:00:00	0.992	0.995	1.012	1.000
6:00:00	0.999	1.004	0.997	1.000
24:00:00	1.004	1.018	0.978	1.000
	0.01			
0:00:00	0.133	0.128	0.138	0.133
1:00:00	0.427	0.418	0.415	0.420
2:00:00	0.614	0.624	0.628	0.622
3:00:00	0.701	0.689	0.667	0.686
6:00:00	0.684	0.685	0.683	0.684
24:00:00	0.788	0.785	0.775	0.783
	0.1			
0:00:00	0.125	0.139	0.13	0.131
1:00:00	0.393	0.387	0.382	0.387
2:00:00	0.548	0.553	0.551	0.551
3:00:00	0.58	0.569	0.568	0.572
6:00:00	0.526	0.528	0.535	0.530
24:00:00	0.535	0.538	0.509	0.527
	1			
0:00:00	0.099	0.126	0.124	0.116
1:00:00	0.342	0.322	0.316	0.327
2:00:00	0.356	0.361	0.356	0.358
3:00:00	0.279	0.286	0.284	0.283
6:00:00	0.218	0.212	0.2	0.210
24:00:00	0.223	0.21	0.241	0.225

Table S4 The best-fit values of response curves and raw data related to Figure 4

Related to Figure 5B

log(inhibitor) vs. response (three parameters)

Best-fit values	EGFP-SD	EGFP-IKZF	EGFP-ZFP91
Bottom	0.05422	1.103	1.006
Top	1.084	1.441	1.185
LogDC50	-3.152	-1.536	-1.679
DC50	0.0007044	0.02913	0.02095
Span	1.029	0.3379	0.1793
95% CI (profile likelihood)			
Bottom	0.03545 to 0.07295	1.055 to 1.144	0.9821 to 1.027
Top	1.059 to 1.108	1.402 to 1.481	1.163 to 1.208
LogDC50	-3.442 to -2.973	-1.854 to -1.187	-1.985 to -1.321
DC50	0.0003615 to 0.001064	0.01399 to 0.06503	0.01035 to 0.04779
Goodness of Fit			
Degrees of Freedom	9	9	9
R squared	0.9986	0.9615	0.9563
Sum of Squares	0.003251	0.008336	0.002707
Sy.x	0.01901	0.03043	0.01734
Number of points			
# of X values	12	12	12
# Y values analyzed	12	12	12

Related to Figure 5D

log(inhibitor) vs. response (three parameters)

Best-fit values	EGFP-SD POM	EGFP-SD AVA	EGFP-SD IBR	EGFP-SD MZG	EGFP-IKZF POM	EGFP-IKZF AVA	EGFP-IKZF IBR	EGFP-IKZF MZG
Bottom	0.01974	0.01996	0.02363	0.0126	0.36	0.07112	0.36	0.385
Top	0.3093	0.3043	0.3096	0.3097	0.4459	0.4411	0.4459	0.4519
LogDC50	-2.853	-2.1	-3.579	-3.875	-1.6	-0.9014	-1.6	-1.952
DC50	0.001402	0.007944	0.0002636	0.0001333	0.02514	0.1255	0.02514	0.01117
Span	0.2896	0.2843	0.286	0.2971	0.08592	0.37	0.08592	0.0669
95% CI (profile likelihood)								
Bottom	0.01682 to 0.02265	0.01292 to 0.02694	0.01784 to 0.02940	0.009963 to 0.01523	0.3424 to 0.3748	0.05317 to 0.08815	0.3424 to 0.3748	0.3796 to 0.3902
Top	0.3050 to 0.3137	0.2971 to 0.3115	0.3002 to 0.3191	0.3053 to 0.3140	0.4317 to 0.4618	0.4325 to 0.4497	0.4317 to 0.4618	0.4455 to 0.4588
LogDC50	-2.885 to -2.821	-2.168 to -2.033	-3.689 to -3.484	-3.952 to -3.808	-2.059 to -1.112	-0.9835 to -0.8179	-2.059 to -1.112	-2.170 to -1.725
DC50	0.001303 to 0.001510	0.006793 to 0.009274	0.0002049 to 0.0003281	0.0001117 to 0.0001557	0.008724 to 0.07735	0.1039 to 0.1521	0.008724 to 0.07735	0.006765 to 0.01884
Goodness of Fit								
Degrees of Freedom	12	12	12	12	10	10	10	10
R squared	0.9992	0.9973	0.9963	0.9993	0.9069	0.9963	0.9069	0.9737
Sum of Squares	0.000146	0.0005509	0.0006764	0.000143	0.001494	0.0008041	0.001494	0.000232
Sy.x	0.003488	0.006775	0.007508	0.003451	0.01222	0.008967	0.01222	0.004817
Number of points								
# of X values	15	15	15	15	15	15	15	15
# Y values analyzed	15	15	15	15	13	13	13	13

Related to Figure 5E

log(inhibitor) vs. response (three parameters)

Best-fit values	EGFP-SD POM	EGFP-SD AVA	EGFP-SD IBR	EGFP-SD MZG	EGFP-IKZF POM	EGFP-IKZF AVA	EGFP-IKZF IBR	EGFP-IKZF MZG
Bottom	0.06229	0.06758	0.04851	0.0164	0.1316	0.1373	0.2424	0.1226
Top	0.9533	0.9484	0.9547	0.9547	1.231	1.246	1.242	1.242
LogDC50	-2.993	-2.187	-4.174	-4.187	-2.292	-1.87	-3.194	-3.265
DC50	0.001016	0.006495	0.00006703	0.00006508	0.005108	0.01349	0.0006392	0.000543
Span	0.891	0.8809	0.9062	0.9383	1.1	1.108	0.9994	1.12
95% CI (profile likelihood)								
Bottom	0.04447 to 0.08000	0.05296 to 0.08212	0.03649 to 0.06052	0.006150 to 0.02664	0.09491 to 0.1678	0.1005 to 0.1734	0.2077 to 0.2768	0.09276 to 0.1523
Top	0.9260 to 0.9805	0.9328 to 0.9642	0.9347 to 0.9746	0.9377 to 0.9717	1.190 to 1.273	1.213 to 1.279	1.187 to 1.297	1.195 to 1.290
LogDC50	-3.067 to -2.919	-2.234 to -2.141	-4.419 to -4.012	-4.384 to -4.047	-2.392 to -2.194	-1.953 to -1.785	-3.317 to -3.077	-3.364 to -3.172
DC50	0.0008571 to 0.001204	0.005834 to 0.007222	3.810e-005 to 9.730e-005	4.126e-005 to 8.979e-005	0.004057 to 0.006399	0.01115 to 0.01639	0.0004816 to 0.0008370	0.0004327 to 0.0006724
Goodness of Fit								
Degrees of Freedom	11	12	12	12	12	12	12	12
R squared	0.9971	0.9987	0.9984	0.9989	0.9946	0.9957	0.9897	0.9938
Sum of Squares	0.00506	0.002516	0.003009	0.00219	0.01635	0.01313	0.02283	0.0172
Sy.x	0.02145	0.01448	0.01584	0.01351	0.03691	0.03308	0.04362	0.03786
Number of points								
# of X values	15	15	15	15	15	15	15	15
# Y values analyzed	14	15	15	15	15	15	15	15

Best-fit values	EGFP-ZFP91 POM	EGFP-ZFP91 AVA	EGFP-ZFP91 IBR	EGFP-ZFP91 MZG
Bottom	0.1492	0.1629	0.1665	0.03183
Top	0.8864	0.8945	0.8854	0.8869
LogDC50	-2.052	-1.296	-3.079	-3.343
DC50	0.008879	0.05054	0.000834	0.0004541
Span	0.7372	0.7315	0.7189	0.8551
95% CI (profile likelihood)				
Bottom	0.1343 to 0.1640	0.1402 to 0.1851	0.1486 to 0.1842	0.02371 to 0.03994
Top	0.8715 to 0.9013	0.8815 to 0.9076	0.8575 to 0.9132	0.8738 to 0.9000
LogDC50	-2.105 to -1.998	-1.362 to -1.232	-3.161 to -2.997	-3.379 to -3.307
DC50	0.007851 to 0.01004	0.04349 to 0.05857	0.0006905 to 0.001006	0.0004174 to 0.0004928
Goodness of Fit				
Degrees of Freedom	12	12	12	12
R squared	0.9982	0.9977	0.9949	0.9992
Sum of Squares	0.002448	0.002709	0.005854	0.001299
Sy.x	0.01428	0.01502	0.02209	0.01041
Number of points				
# of X values	15	15	15	15
# Y values analyzed	15	15	15	15

Table S5 The best-fit values of response curves related to Figure 5

Oligonucleotides

REAGENT or RESOURCE	SOURCE	IDENTIFIER
Oligonucleotides		
gRNA: mCRBN gRNA1: ACAGAUCUUGCACUGGGCAA	This paper	N/A
DNA sequence: ssDNA for homologous recombination of Crbn: CTTTTTTTGTGGCTGTAAATTTCCATCCAATATGGCTTGCACAGATCTTGCACTGGGCAACTGTCCATGCATACCTATAAAATGAAGGAAAGATATAAGTTAAAACCTATTTTTAGCCTTTGTACT	This paper	N/A
Primer: Crbn forward for sequencing: TGAGGTTAAAGCTGGAGCCA	This paper	N/A
Primer: Crbn reverse for sequencing: CAGAGCGAGTTAAGCCCCAA	This paper	N/A
Primer: Crbn forward for I391V allele: ATTTTATAGGTATGCATGGACA(L)G(L)T	This paper	N/A
Primer: Crbn forward for wt allele genotyping: ATTTTATAGGTATGCATGGAC5(L)A(L)T	This paper	N/A
Primer: Crbn reverse for both allele genotyping: GCAAATGCTGTAACCCACTA	This paper	N/A
gRNA: CRBN KO1 gRNA: CGUCAUGCAAAGUUCUCCCG	This paper	N/A
gRNA: CRBN KO3 gRNA: GCAUGACGACGACAGCUGCC	This paper	N/A
Primer: CRBN KO genotyping F: AGAGTTTGATGGAGGCATGCT	This paper	N/A
Primer: CRBN KO genotyping R: GTTGGCTTTAGCTGTCGCAC	This paper	N/A
gRNA: Pdcd1 genome editing gRNA: GCUGAAGAAUCUGGUCAAAG	This paper	N/A
DNA sequence: ssDNA for homologous recombination of Pdcd1: AGACATGAGGATGGACATTGTTCTTGGCCTCTTcataagcgaagccataccggcgaacgcccgttacaatgtgagatctgtggatttacttgtcgacagaaaggtaacctcctccgtcatattaaactgcacacgggggaaaaaccttttaagtgtcacctctgcaacTGACCAGATTCTTCAGCCATTAGCATGCTGCA	This paper	N/A
Primer: Pdcd1 forward for genotyping: GAGAAGACACCAGAGCTCCCTA	This paper	N/A
Primer: Pdcd1 reverse for genotyping: GAAGTGCCCAACAGTAGGATTC	This paper	N/A

Table S6 List of oligonucleotide sequences used in this study
